# Quantum Ghost Spectroscopy Reveals Hidden Coherence in Molecular Aggregates

**DOI:** 10.1021/jacs.6c10272

**Published:** 2026-10-26

**Authors:** Mingran Zhang, Yihe Xu, Vladislav V. Yakovlev, Zhedong Zhang

**Affiliations:** † Department of Physics, 53025 City University of Hong Kong, Kowloon 999077, Hong Kong SAR; ‡ Department of Biomedical Engineering, 14736 Texas A&M University, College Station, Texas 77843, United States; § Shenzhen Research Institute, City University of Hong Kong, Shenzhen 518060, China; ⊥ MOE Key Laboratory for Biomedical Photonics, Wuhan National Laboratory for Optoelectronics, Huazhong University of Science and Technology, Wuhan, Hubei 430074, China

## Abstract

Ultrafast spectroscopy of complex molecules has been achieved under light–matter coupling by the use of laser pulses and single photons. Yet, the excited-state dynamics essential to photochemistry is suffering from the convolution of multiple relaxation pathways and limited resolution in nonlinear signal, which still remain challenging. To address these, we develop time-resolved quantum ghost spectroscopy (tr-QGS) with entangled light, which enables a background-free detection of excited-state emission while offering precise control over temporal resolution. We apply this approach to perylene bisimide (PBI-1) trimers by combining a quantum description of light-molecule interaction with time-dependent density matrix renormalization group (TD-DMRG) simulations. This explicitly includes five vibrational modes and nonadiabatic coupling between electronic states. Our simulations reveal that tr-QGS captures electronic coherence oscillating at 0.6 eV for ∼50 fs, a signature of nonadiabatic coupling that was obscured in time-resolved measurements. Moreover, we observe a direct transfer from electronic to vibrational coherence at 200 fs, providing real-time visualization of vibronic relaxation pathways. Photon entanglement eliminates background noise and ground-state bleaching artifacts, which plague time-resolved measurements. These results establish tr-QGS as a transformative tool for interrogating nonadiabatic dynamics in molecular aggregates, light-harvesting complexes, and photocatalysts, offering a route to reveal quantum coherence effects in chemistry with unprecedented precision.

## Graphical abstract

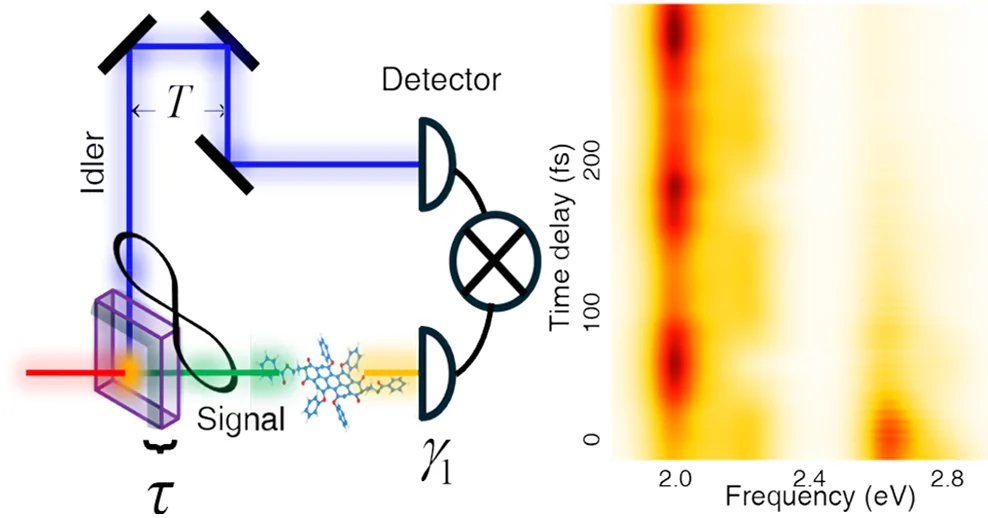

## Introduction

In chemical systems such as light-harvesting complexes, molecular semiconductors, and photocatalytic materials, electronic and vibrational motions are strongly coupled because nuclei and electrons move on comparable ultrafast time scales after photoexcitation. Such coupling gives rise to vibronic transitions, i.e., transitions involving simultaneous changes in electronic and vibrational states which play a central role in determining how energy and charge are absorbed, transferred, converted, and dissipated.^1–7^ Quantum dynamics, as an essential component in biochemical processes and photochemical reactions, carries core information about energy transfer between electrons and nuclei in reaction processes. Ultrafast spectroscopy offers a powerful means of mapping these real-time processes into optical signals. Conventional schemes based on classical light and fluorescence emission detection encounter inherent bottlenecks when applied to excited-state dynamics and ultrafast processes of molecules, e.g., background noise of measurements in light of the signal-to-noise ratio (SNR).^8–11^

Many approaches have been developed, e.g., laser pump–probe schemes and Raman spectroscopy ,whose signal intensities can be improved through the increase of photon flux. Undesirable processes such as higher-order effects (AC Stark shifts, Auger recombination, etc.) and ionization would, however, distort the signals. Additionally, ground-state bleaching (GSB)—an inherent feature of pump–probe schemes—can spectrally overlap with and complicate the interpretation of excited-state processes, particularly when pathway selectivity is limited.^12–15^ Entangled light spectroscopy offers a powerful strategy for resolving molecular structures and relaxation processes. Time–energy correlations have been used to image cellular and tissue structures, with incredibly enhanced sensitivity.^16–18^ Recent experiments demonstrated the capability of measuring molecular absorption using entangled light, indicating internal energy conversion.^19,20^ Despite these advancements, ultrafast spectroscopy with entangled light is still facing obstacles.

One of the main challenges is the knowledge gap between molecular spectroscopy and entangled photons, even at the theoretical level. Normally, calculations of molecular excited states, as achieved by quantum chemistry methods, provide a complete set of electronic and nuclear degrees of freedom and the underlying full-dimensional dynamics. Spectroscopy, however, can access only a portion of the information about excited-state structures, yielding a projection of molecular dynamics. Several studies of ultrafast spectroscopy and excited-state structures have been reported, e.g., for polymethine^21–24^ and rylene dyes,^25–28^ which employed a classical description of light fields. These may smear out advanced information such as interference and energy fluctuations, which underlie the essential development of new spectroscopic techniques using quantum states of light.

In this theoretical study, we develop a time-resolved quantum ghost spectroscopy (tr-QGS) with entangled light to monitor excited-state relaxation and signatures of coherence associated with exciton transfer and internal conversion (Figure 1a,1b). The unique properties of time–energy correlations between the photons bring out novel joint-detection-based methodology for molecular fluorescence, e.g., yielding high sensitivity and enabling real-time monitoring of single-photon spectroscopic signals in biological membranes.^19,20^ Beyond the nanosecond scale realized in experiments, our results demonstrate an achievable scheme to acquire femtosecond dynamics of PBI aggregates, e.g., electronic coherence of highly excited states within 50 fs and vibrational coherence on longer time scales of ∼300 fs. These can be obscured in transient absorption measurements for some condensed-phase molecules when their spectral features overlap with the GSB component. The tr-QGS includes the emission process only, providing an alternative pathway to reveal coherence dynamics in a time-energy-resolved manner. We further develop a time-dependent density matrix renormalization group (TD-DMRG)^29–32^ approach for the tr-QGS. The TD-DMRG allows us to involve low-frequency vibrations as baths in a rigorous and efficient way. The tr-QGS requires the off-diagonal components of the time propagation of excited states, i.e., the phases of the time-evolution operator, rather than the diagonal components in the calculation of the absorption spectrum. Our results offer a scheme for ultrafast spectroscopy using quantum technology, which is feasible in experiments and provides a multidimensional probe of molecular structures.

**Figure 1:**
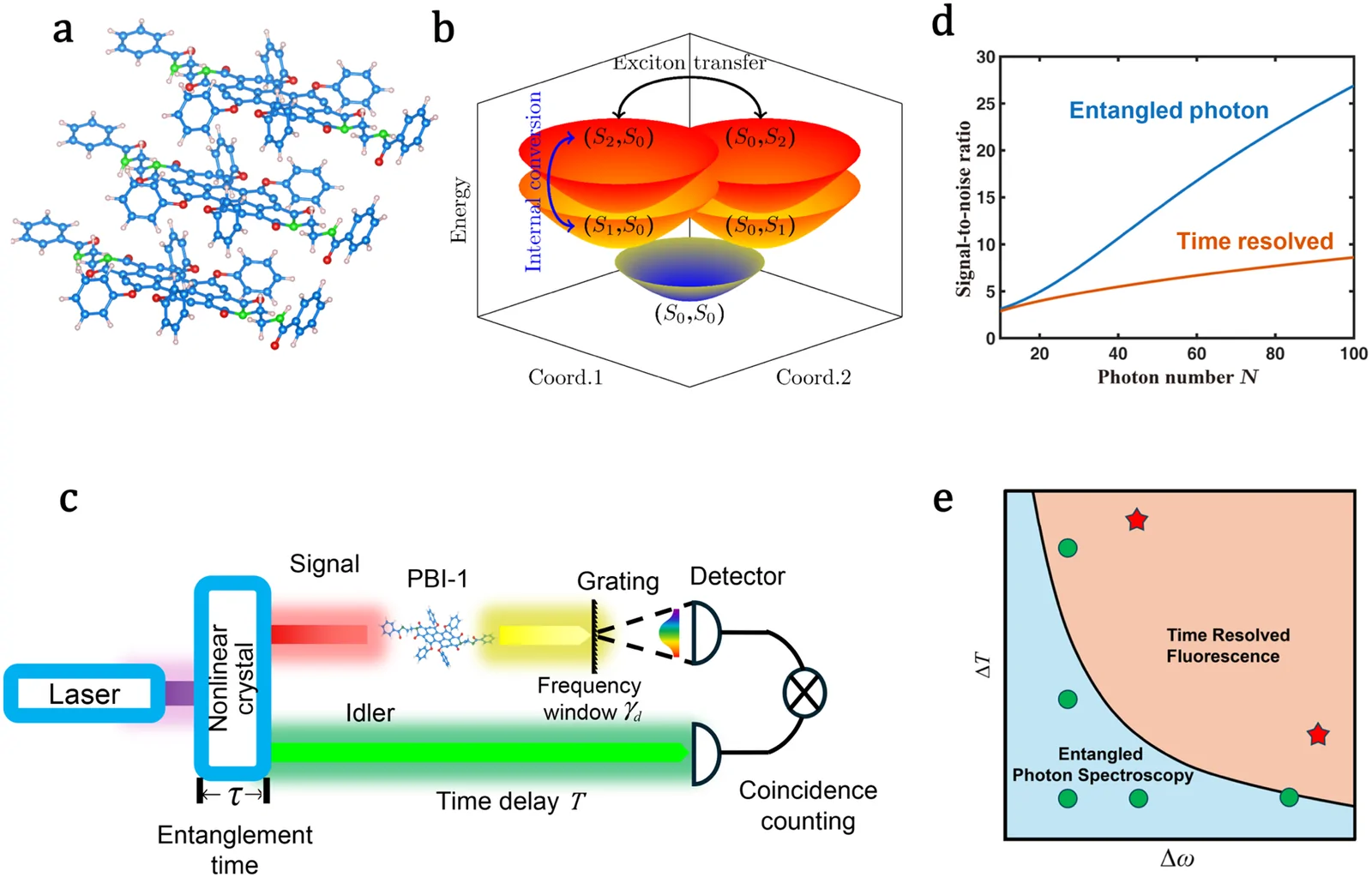
Entangled-light spectroscopy. (a) Molecular structure of the PBI-1 trimer. (b) Schematic diagram of the involved PESs of the PBI-1 molecule. (*e*_1_,*e*_2_) denotes a pair of nearest-neighbor monomers, one is in state *e*_1_ and the other is in *e*_2_. Coordinates 1 and 2 are the same vibrational mode inside monomers 1 and 2, respectively. (c) Proposed experimental setup. A pump pulse enters a nonlinear crystal to generate two light beams *s* (signal) and *i* (idler) which are entangled with an entanglement time τ. s-arm photons interact with molecules whereas i-arm photons propagate freely with a time delay *T*. With respect to *s*-arm photons, molecules emit fluorescence which is collected by an energy-resolved detector with spectral resolution γ*_d_*, yielding photon-coincidence counting that gives the optical signal. (d) Signal-to-noise ratio calculated for using entangled photons (blue) and lasers (orange); the entangled-photon scheme exhibits linear scaling. (e) Fisher-information-based variances of time and energy scales calculated for laser spectroscopy and quantum-light measurements. Green spots are for tr-QGS using entangled light in Figures 4 and 5, and red stars are for time-gated fluorescence in Figure 6.

## Results and Discussion

### Quantum Dynamics

The PBI and its derivatives are well known for their visible-light absorption, electron-accepting ability, and stability, which have attracted numerous efforts in optoelectronic devices and energy-transfer cascades.^33–36^ These properties are owing to PBI derivatives’ characteristic molecular architectures. The planar perylene core allows the formation of aggregates which are stabilized by intermolecular hydrogen bonds. This J-type stacking causes a significantly strong light-harvesting ability and a high quantum yield.^37^ Here we study the tr-QGS for the exciton process in PBI-1 trimers in a dilute solvent whose molecular structure and potential energy surfaces are shown in Figure 1a and 1b, respectively. In each monomer, two electronically excited states are considered, i.e., |S_1_⟩ and |S_2_⟩. We calculate the excited-state dynamics with the TD-DMRG method incorporating the molecular Hamiltonian in eq 8. The TD-DMRG dynamics is essentially outlined in Methods for the simulation approach. The main results of the TD-DMRG dynamics are shown in Figure 2. We first investigate the population and coherence dynamics of the PBI-1 dimers, which are described by the diagonal and off-diagonal elements of the reduced density matrix ρ̂, i.e.,

1 $$\mathrm{\rho ̂}\left(t\right)=\left(\begin{array}{cc} \rho_{S_{1},S_{1}}\left(t\right) & \rho_{S_{1},S_{2}}\left(t\right)\\ \rho_{S_{2},S_{1}}\left(t\right) & \rho_{S_{2},S_{2}}\left(t\right) \end{array}\right)$$

**Figure 2:**
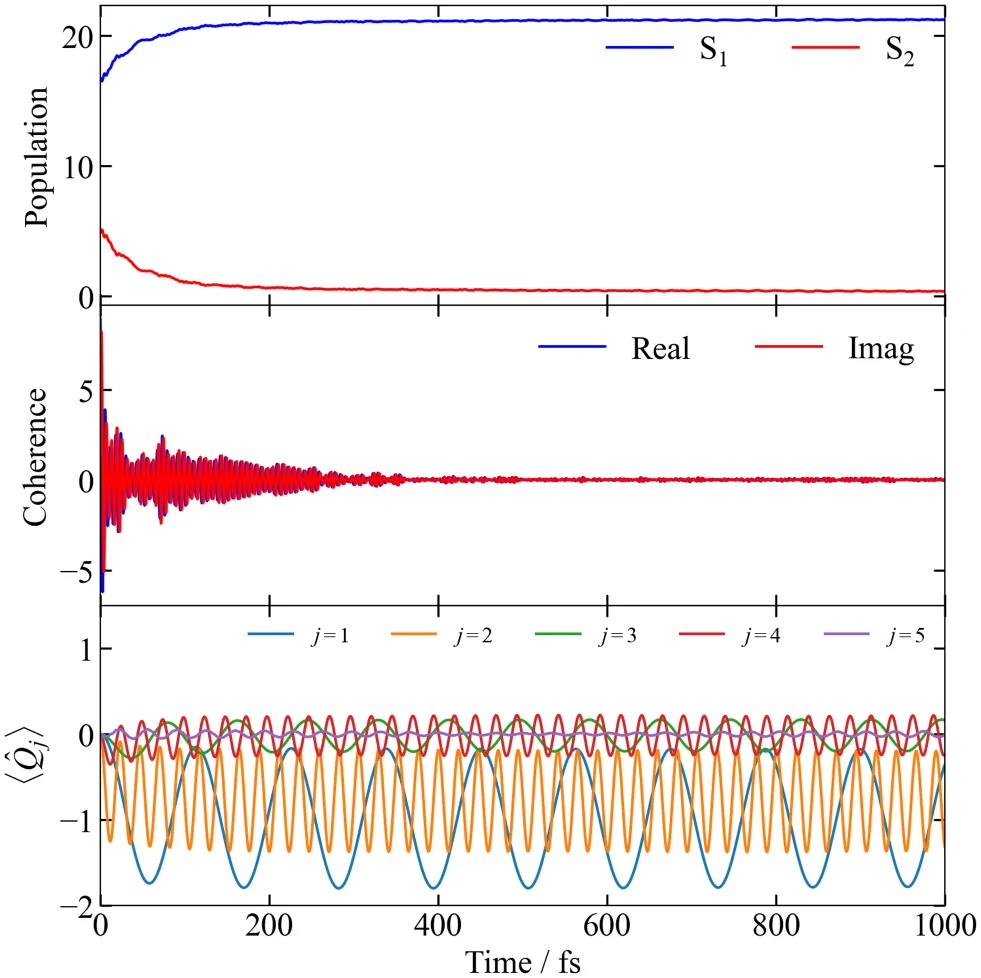
Quantum dynamics of PBI-1 trimers. (a) Populations at S_1_ and S_2_ states. (b) Electronic coherence $\rho_{S_{1},S_{2}}$ . (c) Mean dimensionless position of the *j*th vibrational mode. The evolution time is set to 1000 fs with a step of 0.166 fs.

The populations of S_1_ and S_2_ are shown in Figure 2a. We find that the transition from S_2_ to S_1_ dominates the first ∼150 fs of the dynamics, and the aforementioned nonadiabatic coupling accounts for this process. The initial ratio of S_1_ and S_2_ is exactly equal to the ratio of the squares of their corresponding transition dipole |*V*|^2^. The populations are relatively smooth because there is no strong and direct coupling between S_1_ and S_2_. These signatures of electronic couplings and their control properties have been highlighted by recent advancements in energy harvesting and migration.^38–42^ However, the populations are insufficient for analyzing the dynamical properties. Therefore, we also show the electronic coherence dynamics in Figure 2b. In contrast to the populations, the coherence is often complex. We can find both the real and imaginary parts of the coherence which oscillate significantly until ∼200 fs and decay afterward. They oscillate at the same frequency, which is about 0.6 eV, close to the energy difference between S_1_ and S_2_. Besides the electronic states, we calculated the vibrational coherence measured by ⟨*Q*_*j*_(*t*)⟩ where *Q* is the coordinate of the vibrational mode. The oscillation frequencies in Figure 2c correspond to those of the normal modes. Here, mode 1 and mode 3 are of low frequency and the others are of high frequency. Moreover, as shown in Figure 2c, vibronic coupling leads to significant shifts of the equilibrium positions of the 5 modes. Notably, we can observe a nonadiabatic signature from the coupling to mode 5 such that a fast oscillation occurs within the first 20 fs and subsequently becomes smoother.

### Time-Resolved Quantum Ghost Spectroscopy

We will focus on the tr-QGS to probe the exciton dynamics in PBI trimers, where *s* photons excite the S_1_ and S_2_ electronic states and *i* photons serve as a reference beam. The emitted fluorescence photons in the *s* arm then coincide with the *i* photons, resulting in a coincidence-counting signal^43,44^

$$S\left(\omega ,T\right)\propto \int_{-X}^{X}dt\left\langle a_{s}^{†}\left(\omega \right)a_{i}^{†}\left(t\right)a_{i}\left(t\right)a_{s}\left(\omega \right)\right\rangle$$ 2

where $a_{i}\left(t\right)=\frac{E_{i}}{2\pi }\underset{\omega^{\prime }}{\sum }a_{i}\left(\omega^{\prime }\right)e^{-i\omega^{\prime }t}$ ; *a*_*s*_(ω) and *a*_*i*_(ω) are the annihilation operators for the photons in the *s* and *i* arms, respectively. The average ⟨···⟩ is taken over the wave function of the joint molecule-light system. Photons in the two arms are entangled in the time–energy domain, arising from spontaneous parametric down-conversion (SPDC) in the χ^(2)^ nonlinear crystals (Figure 1c). In fact, eq 2 does account for the effect of the slow instrumental response of the single-photon detectors, i.e., X∼2 ns.

After electronic excitation by *s* photons, the *i* photons arrive later, forming a temporal gate for the *s*-arm fluorescence. The quantum correlation ensures a well-defined time delay *T* between *s* and *i* photons that can be varied, thereby generating the tr-QGS spectrum. To this end, the molecule-light dipolar interaction has to be involved, to imprint molecular information into *S*(ω, *T*). The fluorescence photons render the quantum field of *s*-arm photons, at the few-photon level. The fourth-order perturbative expansion can thus be carried out for eq 2, and one obtains (see Methods)­

3 $$S\left(\omega ,T\right)\propto \int_{-\infty }^{\infty }\left[d\tau \right]\chi \left(t_{4},t_{3},t_{2},t_{1}\right)C\left(\omega ;t,t_{4},t_{3},t_{2},t_{1}\right)$$

where χ­(*t*_4_, *t*_3_, *t*_2_, *t*_1_) is the molecular response function given by correlations of dipole operators; $C\left(\omega ;t,t_{4},t_{3},t_{2},t_{1}\right)=e^{i\omega t_{4}}\left\langle \Phi \right|E_{s}^{†}\left(t_{1}\right)E_{s}\left(t_{3}\right)E_{s}^{†}\left(\omega \right)E_{i}^{†}\left(t\right)E_{i}\left(t\right)E_{s}\left(t_{2}\right)\left|\Phi \right\rangle$ is the six-point Green’s function of light, and [dτ] ≡ d*t*d*t*_4_d*t*_3_d*t*_2_d*t*_1_. For spectroscopy using lasers, the optical fields are classical so the *C* function is a product of complex functions. For spectroscopy with entangled light, nevertheless, the optical fields have to be treated quantum mechanically, i.e., on an equal footing with the molecular excited states.^6^ This leads to a field Green’s function distinct from the one for lasers. Therefore, the wave function of entangled twin photons is needed and it reads

$$\Phi \left(\omega_{s},\omega_{i}\right)=Q\exp[-{\left(\omega_{s}+\omega_{i}-\omega_{+}\right)}^{2}\frac{1}{\sigma^{2}}]\times \exp\left[-{\left(\omega_{s}-\omega_{i}-\omega_{-}\right)}^{2}\frac{\tau^{2}}{4}\right]$$ 4

where ω_+_ = 4.7 eV (wavelength at 269 nm) is the central frequency of the pump field acting on the crystals, e.g., β-barium borate (BBO); ω_–_ = 0.7 eV results from the dispersion inside the crystals, and *Q* is a normalization constant. The first Gaussian function in eq 4 is from energy conservation in χ^(2)^ process. Normally, σ is small, i.e., σ ≪ 1/τ so that *s* and *i* photons are spectrally anticorrelated, as depicted in Figure 3. Here, we set σ^–1^ = 400 fs in the simulations. The Gaussian function exp­[−(ω_*s*_ – ω_*i*_ – ω_–_)^2^τ^2^/4] is broadband so as to precisely control the delay *T* at a variance τ; τ is the so-called entanglement time governed by the group velocity of the light waves propagating inside the nonlinear crystals. Figure 3a and 3b depicts the spectral correlation of photons as a function of τ. It turns out that the time–energy correlation is eroded by longer τ. The full Hamiltonian consists of the molecular Hamiltonian in eq 8 and the dipolar interaction with entangled twin photons.

**Figure 3:**
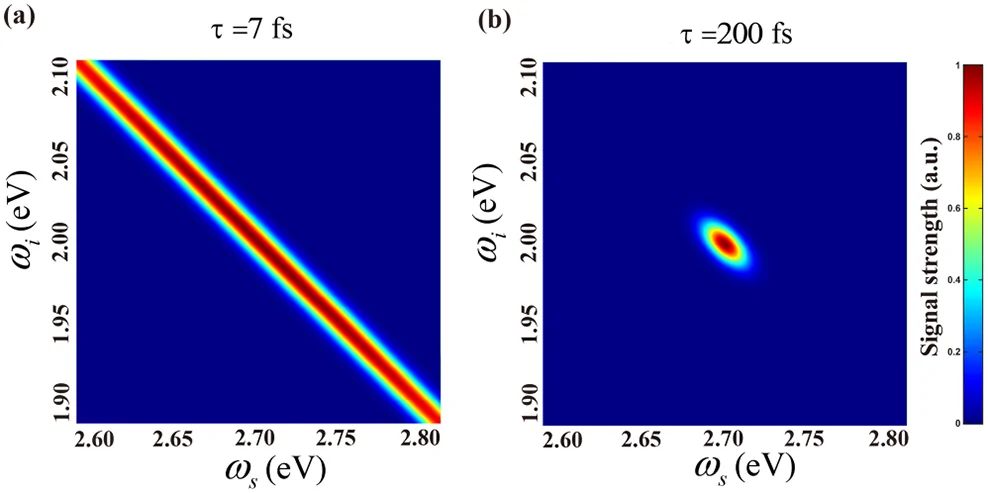
The entangled-photon wave function |Φ­(ω*_s_*, ω*_i_*)| from eq 4 reveals the quantum correlation between *s* and *i* photons. (a) Entanglement time τ = 7 fs; (b) τ = 200 fs.

It is worth noting that the pump field acting on the χ^(2)^ crystals can have a narrower bandwidth , namely, σ^–1^ ∼ 1 ns. From an experimental viewpoint, nanosecond (subnanosecond) pulses are more practical than subpicosecond pulses. Simulations of tr-QGS using σ^–1^ = 1 ns do not present a significant difference from those with σ^–1^ = 400 fs (Figure 3a), as shown in Supporting Information (SI). This demonstrates the flexibility of opting laser pulses for generating entangled photons tailored to the tr-QGS.^19^

The use of entangled light is capable of suppressing the background noise, e.g., intensity fluctuations. In particular, coincidence counting is a *g* ^(2)^-level measurement, which requires identical numbers of photons in the *s* and *i* arms, thus eliminating the background noise.^45^ However, the intensity of a laser normally follows a Poisson distribution, resulting in fluctuations $∼\sqrt{n_{photon}}$ . By further involving the photon loss, the photon distribution function is calculated for achieving the SNR for *N* entangled-photon pairs. We demonstrate a nearly linear scaling of the SNR with the photon number, as shown in Figure 1d. For more details, refer to SI.

The tr-QGS combines the molecular optical response and photon correlation functions, as derived in the SI using time-dependent perturbation theory with the molecule-light dipolar interaction. As the pump field for generating the entangled photons is narrow-band, the first Gaussian function in eq 4 is temporally smooth and thus allows the slowly varying approximation, as shown in the SI. Therefore, some algebra leads from eq 3 to tr-QGS

$$S\left(\omega ,T\right)\propto \frac{1}{\tau }\times \underset{\nu }{\sum }{|\int_{0}^{\infty }dte^{iE_{0}t}\left\langle \Psi_{0}^{\left(\nu \right)}\right|V\left(\omega \right)U\left(t\right)V^{†}\left|\Psi_{0}^{\left(0\right)}\right\rangle M\left(\frac{T-t}{\tau }\right)|}^{2}$$ 5

where $\left|\Psi_{p}\left(t\right)\right\rangle =U\left(t\right)V^{†}\left|\Psi_{0}^{\left(0\right)}\right\rangle$ is the real-time wave function containing full information about the excited states at which we are targeting; $\Psi_{0}^{\left(\nu \right)}=\psi_{0}\left(q-Q\right)\chi_{\nu }\left(Q\right)$ denotes the electron-vibration wave function at the electronic ground state, and **ν** ≡ {ν_1_, ν_2_, ···}. The electronic ground and excited states are calculated by multireference configuration interaction (MRCI)^46^ as introduced in Methods. *χ*_**ν**_(**Q**) is the vibrational wave function having ν_*k*_ occupation number at the *k*th mode with ν_*k*_ ≠ 0, and *E*_0_ is the ground-state energy. The time-gating function M is the Fourier component of the broadband Gaussian function exp­[−(ω_*s*_ – ω_*i*_ – ω_–_)^2^τ^2^/4] in eq 4. $V\left(\omega \right)=\int dt^{\prime }e^{i\omega t^{\prime }-\gamma \left|t^{\prime }\right|}V\left(t^{\prime }\right)$ projects the wave function |Ψ_p_(*T*)⟩ into the Fourier domain and extracts its spectral component, thereby generating the spectral lines; V denotes the dipole operator. $e^{-\gamma |t^{\prime }|}$ gives the decay of vibronic states, with γ = γ_0_ + γ_*d*_ where $\gamma_{0}^{-1}∼10$ fs is the intrinsic decay rate typically for PBI-1 trimers, and γ_*d*_ measures the bandwidth of *s*-photon detector, e.g., arising from an imperfect grating. Both τ and γ_*d*_ are tunable in experiments.

From eq 5, the PBI-1 dynamics are described by the time-evolution operator U­(*t*), which is gated by entangled light. The electric dipole V­(ω) generates spectral lines subject to dephasing and detector bandwidth. The tr-QGS is thus time-energy-resolved. All these matrix elements are obtained from the molecular Hamiltonian in eq 8, whose eigenfunctions can be computed with TD-DMRG using the Kylin-V software package.^47^

With the response functions calculated from TD-DMRG results, we applied eq 5 for simulating the tr-QGS and probing the excited-state dynamics illustrated in Figure 1c. As shown in Figure 4a, the energy scale is dominated by the decay of coherence γ_0_ = 66 meV and the time scale is subject to the arrival time of th ephoton pair that has a precision τ (gating parameter) arising from the temporal correlation of photons. Here, we suppose the detector has ideal frequency resolution γ_*d*_ = 0 so that γ = 66 meV. The simulated spectroscopic signal reveals the populations that directly reflect the underlying excited-state energy transfer. Specifically, the amplitude of the peak associated with the S_1_ state (around 2.0 eV) exhibits an increase, and S_2_ (near 2.67 eV) decreases within the first 150 fs. This reveals an internal conversion from S_2_ to S_1_ state, along with the quantum dynamics calculations depicted in Figure 2a. Afterward, the ratio of the S_1_ and S_2_ populations remains nearly unchanged. More importantly, the signal reveals a splitting of the S_1_ state of PBI-1 trimers into two sublevel components at 2.0 and 2.2 eV, respectively. In Figure 4a, as the delay time varies, both spectral lines show oscillations at a period >100 fs (∼300 cm^–1^, close to the frequency of vibrational mode 1 as seen in Figure 2c) but with opposite phases. This evidences the vibrational coherence of mode 1 in all split S_1_ states and of exciton transfer between the monomers. The S_2_ peak shows a rapid oscillation with a period ∼6 fs (∼0.67 eV, close to $E_{S_{2}}-E_{S_{1}}$ )—due to a narrow gate $w=\tau /\sqrt{2}\approx 4$ fs—which is the signature of electronic coherence $\rho_{S_{1},S_{2}}$ . Such a feature cannot be visualized in single-photon molecular fluorescence and absorption.

**Figure 4:**
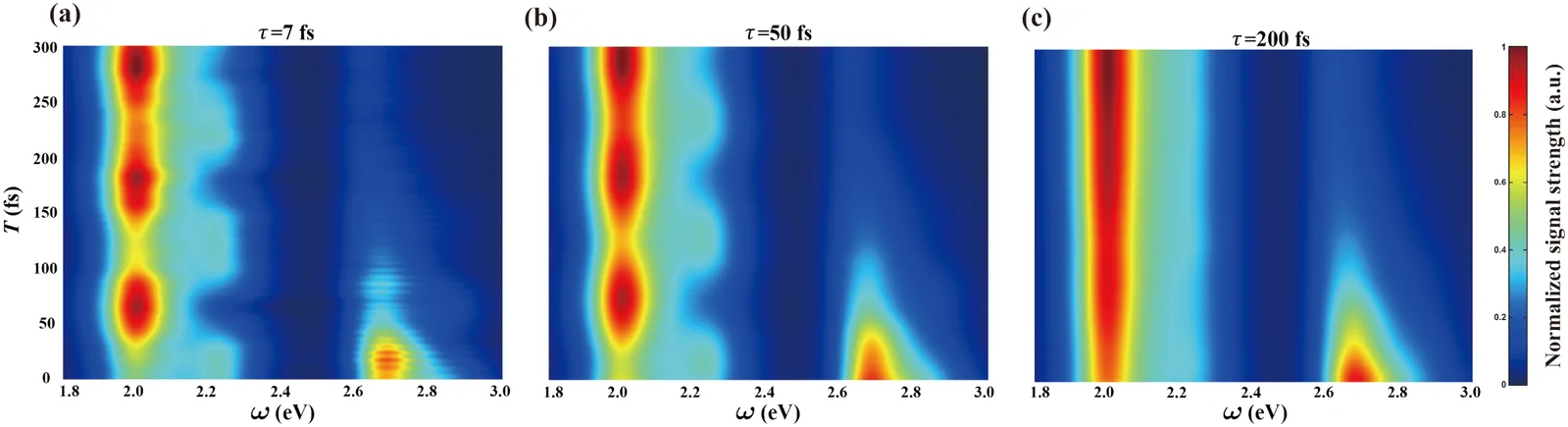
tr-QGS signal with various temporal-gating parameters τ, where ω is the emission frequency and *T* is the delay between *s* and *i* photons. (a) τ = 7 fs; (b) τ = 50 fs; (c) τ = 200 fs. Parameters are γ = 66 meV and ω_+_ = 4.7 eV, in line with recent experiments.^48^ Molecular parameters are reproduced from ref 46. The values of τ correspond to the three green circles aligned horizontally in Figure 1(e). All signals at ω > 2.6 eV have been rescaled , i.e., 50x.

Our simulations suggest that photon entanglement may resolve oscillations as fast as ∼6 fs (Figure 4a), a regime that would be inaccessible with longer gates (Figure 4b, 4c). While experimental imperfections would likely reduce this resolution, the result illustrates the potential of tr-QGS for probing electronic coherences.

From eq 5, one knows that our scheme is engaged with the third-order optical process. Thus, the signal intensity around the S_2_ state is appreciably lower than the one for S_1_. Therefore, for clear monitoring, it is better to rescale the signals for ω > 2.6 eV. The peaks of the tr-QGS cover several vibronic sidebands, which involve the internal conversion process shown in Figure 1b. The wave packet dynamics of the S_2_ state, at frequencies near 2.67 eV, exhibits a composite structure that comprises the contributions from numerous vibronic states, i.e., at 2.7, 2.73, and 2.78 eV. High-frequency vibrations decay faster than the low-frequency ones. This results in multiple time scales of relaxation dynamics, yielding a diffusion-like profile of the spectral line at 2.67 eV. The initially broad S_2_ signal contains multiple vibronic transitions and then undergoes progressive narrowing as the high-frequency vibrations decay. The rest lines are narrower considerably, when *T* > 50 fs, as low-frequency vibrations dominate.

Eq 5 indicates that the tr-QGS spectrum can be controlled by photon entanglement. By varying the gating parameter τ, the dynamics is interrogated differently. Figure 4 shows a dilution of the temporal resolution. In Figure 4b, the rapid oscillation at ∼2.7 eV disappears and a smoother signal is obtained, when τ = 50 fs. If one further increases τ, the slow oscillations along the S_1_ resonance are smeared out as well, as shown in Figure 4c. Nevertheless, we find that the gating parameter τ does not significantly obscure the frequency-domain information. Even with τ = 200 fs as depicted in Figure 4c, the tr-QGS can still reflect the S_2_ → S_1_ transition when tracing along the delay *T*. But the which-way information and the coherence signatures are erased from the spectrum. This means a control of the temporal scale independent of the spectral one in the emission spectrum, thanks to the entangled photons. Normally, a shorter time window is preferred because of the need for coherence signals that can help visualize nonadiabatic transitions.

The energy resolution of the tr-QGS is mainly determined by two aspects. One is the natural line-broadening of molecules which is intrinsic, regardless of external fields. The other is associated with the *s*-photon detector, which is dominated by the grating period. γ_*d*_ is introduced therein for the width of this energy window. Figure 5 shows the control of frequency scale by γ_*d*_. Compared with the ideal case in Figure 5a, the signal in Figure 5b with an additional 66 meV makes the intensity of the peak at 2.67 eV decreasing. The opposite phases between the 2 and 2.2 eV resonances are also hard to reveal. Moreover, in Figure 5c, the signal with γ_*d*_ = 163 meV only shows a broad band of S_1_ but cannot reveal the split sub-bands and related exciton dynamics. The peak of the S_2_ state is fully covered by the sideband of the S_1_ peak and thus disappears in the spectrum. Therefore, detectors with narrower bandwidth γ_*d*_ can always facilitate to resolve more energy levels and dynamics. Despite the poor spectral resolution, the signals in Figure 5b and 5c still maintain a rapid oscillation of period 6 fs, which reflects the electronic coherence between S_1_ and S_2_. This elaborates the control of the spectral window, independent of the temporal resolution.

**Figure 5:**
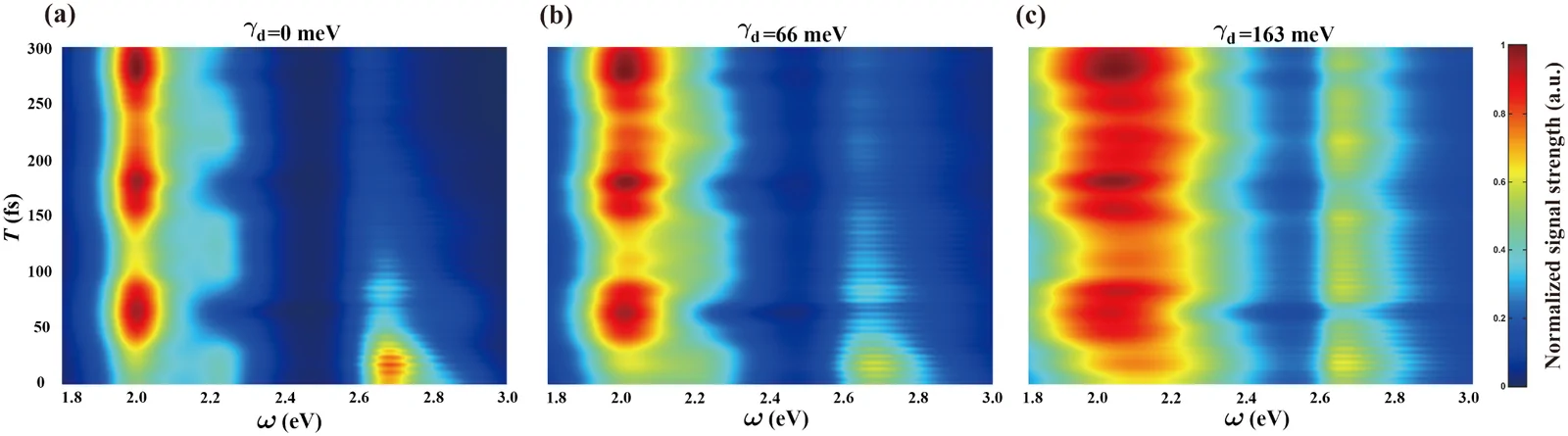
tr-QGS with different spectral-gate parameters. (a) γ*_d_* = 0; (b) γ*_d_* = 66 meV; (c) γ*_d_* = 163 meV. Parameters are τ = 7 fs and γ_0_ = 66 meV. Others are the same as Figure 4. The values of γ*_d_* correspond to the three green circles aligned vertically in Figure 1(e).

### Comparison with Laser Pump–Probe Spectra

The laser pump–probe (LPP) spectra can be obtained from the third-order perturbative series with semiclassical light-matter interaction. The result is $S_{\mathrm{PP}}=R\left(S_{\mathrm{GSB}}+S_{\mathrm{ESE}}\right)$ with

6a $$S_{\mathrm{ESE}}\left(\omega ,T\right)\propto \underset{\nu }{\sum }\int d\tau {|\langle \Phi_{0}^{\left(\nu \right)}|V(\omega )U(\tau )V^{†}\left|\Phi_{0}^{\left(0\right)}\right\rangle |}^{2}E\left(\tau -T\right)Ẽ\left(\omega_{c}-\omega -E_{0}^{\nu }\right)$$

6b $$S_{\mathrm{GSB}}\left(\omega ,T\right)\propto \underset{\nu }{\sum }{|\langle \Phi_{0}^{\left(\nu \right)}|V(\omega )V^{†}\left|\Phi_{0}^{\left(0\right)}\right\rangle |}^{2}e^{iE_{0}^{\nu }T}Z^{\left(\nu \right)}\left(\omega_{c},\omega \right)$$

where $Z^{\left(\nu \right)}\left(\omega_{c},\omega \right)=\int d\omega^{\prime }Ẽ\left(\omega -\omega_{c}-E_{0}^{\nu }\right)Ẽ\left(\omega^{\prime }\right)Ẽ\left(\omega^{\prime }+E_{0}^{\nu }\right)$ is a Gaussian-shaped function. $E\left(t\right)=\exp\left[-\frac{t^{2}}{\sigma^{2}}\right]$ is the envelope of the pump and probe fields, and Ẽ(ω) is the Fourier component of E(t) . The symbol **ν** = {ν_1_, ν_2_, ...} denotes the vibrational excitations, and $E_{0}^{\nu }$ is the energy of $\Phi_{0}^{\nu }$ .

The excited-state emission (ESE) component in eq 6 can reveal the excited-state dynamics of molecules. Notably, two-dimensional electronic spectroscopy (2DES)—as a substantial variation of the LPP—provides a powerful platform for probing population and coherence dynamics, with established strategies for distinguishing different coherence pathways.^38,49–52^ However, it is hard to isolate the ESE from the full pump-probe and 2DES signals. From our molecular model, both the GSB and ESE components contribute substantially to the signal. Knowing that the **ν** = 0 term dominates in eq 6 and 7, the GSB and ESE remain positive resonances and cannot be separated spectrally. Such a spectral overlap complicates the analysis of the dynamics. In particular, the two components may merge into a single broad band; the ESE intensity could be overestimated due to the coincidence with the GSB component.

Unlike the LPP and 2DES, the QGS demonstrates the capability of suppressing GSB contamination, as seen from eq 5. This would be promising for the study of the photochemical processes. Recently, extensive efforts were devoted to the exploration of nonlinear spectroscopy with entangled light and squeezed light, toward higher SNR and resolution.^53,54^ Selectively accessing different terms of molecular correlation functions, nevertheless, still remains challenging. Therefore, the QGS maintains unique advantages of higher selectivity in conjunction with independent control of the time-energy resolutions.

### Time-Gated Fluorescence Spectrum

In conventional schemes, the time-resolved single-photon fluorescence imprints the molecular excited states by applying time and frequency gates; the molecules are excited by a laser pulse. Although the time and frequency gates can be controlled individually, the fluorescence signal shows a joint time-frequency scale σ̃_*ω*_σ̃_*t*_ ⩾ 1 where σ̃_*ω*_ and σ̃_*T*_ are the time and frequency scales of the two gates. Detailed derivations are provided in the SI. Figure 6a shows the time-gated fluorescence signal that has a short temporal gate but smears out the molecular excited states. The temporal scale ${\mathrm{\sigma ̃}}_{T}=\sigma_{T}^{-1}+\Omega^{-1}=7\mathrm{fs}$ coincides with the gating parameter τ of tr-QGS, where $\sigma_{T}^{-1}=3.5\mathrm{fs}$ is the temporal gate width and Ω^–1^ = 3.5 fs is the duration of the pump laser. Moreover, for a frequency scale σ_ω_ = 40 meV close to the one using entangled photons (Figure 4a), the spectrum exhibits good spectral but poor temporal resolution, as depicted in Figure 6b where the interplay between S_1_ and S_2_ as well as the vibronic coherence are smeared out. Nevertheless, the tr-QGS with imperfect parameters is still capable of reflecting the real-time dynamics of molecular states. In particular, the two sublevels of S_1_ at 2.0 and 2.2 eV, which can be visualized in Figure 4c, are merged into a broad peak in Figure 6b.

**Figure 6:**
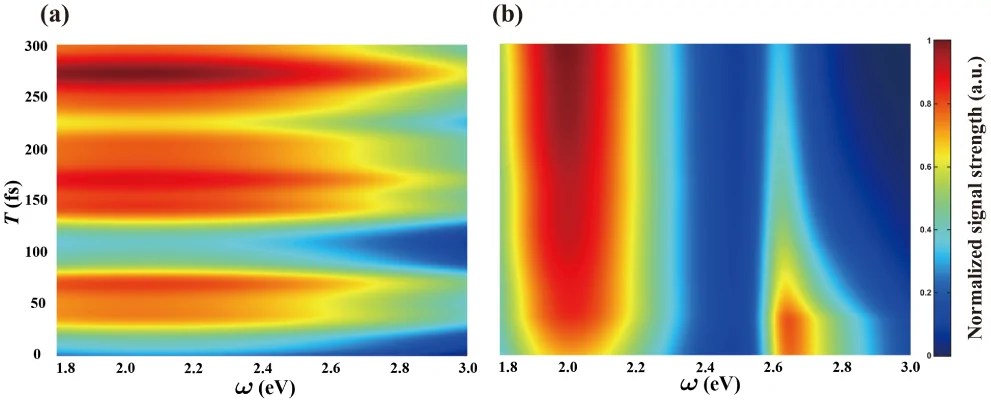
Time- and frequency-gated fluorescence signal. (a) $\sigma_{T}^{-1}=3.5\mathrm{fs}$ and $\sigma_{\omega }^{-1}=0.5\mathrm{fs}$ . The signal has a short temporal gate, yielding a broad resonance that covers both S_1_ and S_2_ states. (b) $\sigma_{T}^{-1}=3.5\mathrm{fs}$ and $\sigma_{\omega }^{-1}=100\mathrm{fs}$ . The signal shows a better spectral scale to illustrate S_1_ and S_2_ but a worse temporal scale that masks the oscillations. The delay *T* is relative to the pump laser pulse. (a) and (b) correspond to the red stars in Figure 1(e).

## Conclusion and Remarks

In addition to the theoretical efforts above, the experimental feasibility of the tr-QGS can be evaluated, so as to provide testable predictions for the implementation of probing nonadiabatic dynamics in molecular aggregates. The entangled twin-photon state described by eq 4 is generated from nonlinear crystals such as BBO. The entanglement time τ that offers a time-gating parameter is governed by the crystal geometry and refractive indices through a time-difference function^55,56^ resulting from the phase-matching condition inside the crystals. This enables the estimation of the crystal size for a given τ. In particular, for τ = 7 fs, one obtains a ∼0.5 mm thickness of BBO crystal (see SI). And the entanglement time can be further varied through different sizes of BBO. Moreover, the pump bandwidth σ^–1^ ∼ 400 fs^–^1 ns, which acts on the BBO, is a standard value achievable in type-I SPDC. This bandwidth shows high flexibility for opting the pump lasers for generating entangled photons tailored for the tr-QGS. This may facilitate to implement the tr-QGS in experiments.

The time delay *T* between the *s* and *i* beams is normally controlled by a motorized delay stage with ⩽1 fs precision, achievable with piezo stages and interferometric stabilization. Spectral resolution is set by a grating spectrometer, e.g., a Czerny–Turner with 1200 l/mm grating, with resolving power λ/δλ ≈ 2000–5000, corresponding to a scale of 0.1–0.4 eV at 500 nm.

In summary, we developed the tr-QGS for probing nonadiabatic molecular dynamics and demonstrated its application to PBI-1 trimers for coherent energy migration. By exploiting the quantum correlations of entangled photon pairs, the tr-QGS enables the capability of accessing excited states and their dynamics. Our simulations showed that the tr-QGS resolved the 0.6 eV electronic coherence while simultaneously distinguishing the vibronic sublevels, which was used to be a hard task. Our results further provide a visualization of electronic-to-vibrational coherence transfer. We observe the real-time conversion from electronic coherence (within the first 50 fs) to vibrational coherence (emerging after 200 fs) in molecular aggregates. This forms a map of the nonadiabatic relaxation pathways followed by vibrational cooling. Finally, our scheme circumvents the GSB and isolates excited-state emission—a capability that is not attainable in conventional pump-probe spectroscopy. And the coincidence counting inherent to the tr-QGS enables operation at low photon fluxes, minimizing the higher-order effects that can distort signals. This is particularly valuable for photochemically sensitive systems, light-harvesting complexes, and single-molecule studies.

It is worth noting the broader outlook of the theoretical approach developed in this work. The ability of monitoring electronic and vibrational coherences may open new routes for understanding conical intersection dynamics. The tr-QGS could capture these coherences , producing a quantum-mechanical “movie” of the intersection events. Moreover, the tr-QGS could be extended to chirality probing and enantio-specific detection, using entangled light with structures of polarization and angular momentum, which may facilitate to address the long-standing challenge in asymmetric photochemistry and pharmaceutical synthesis. By further combining the tr-QGS with plasmonic antennas or dielectric metasurfaces, it would be possible to push the sensitivity down to the single-molecule level, boosting studies of heterogeneous catalysis, single enzymes, and defect centers in low-dimensional materials.

## Methods

### Entangled Twin Photons

Quantum-entangled light is a promising next-generation source for quantum computation^57–59^ quantum information.^60,61^ and quantum sensing.^62–64^ The generation of entangled light beams has been developed into a mature equipment through a nonlinear crystal.^65–67^ As shown in Figure 1c, the incident pump beam is split into signal and idler arms. The beams in the *s* and *i* arms have some internal correlations, e.g., from the conservation of momentum **k**_*p*_ = **k**_*s*_ + **k**_*i*_ for **k**_*j*_ is the wavenumber of light in the *j* arm, and energy conservation ω_*p*_ = ω_*s*_ + ω_*i*_ for the light frequency. Since the beams in both arms are generated simultaneously, their arrival times are equivalent. By applying a time-delay segment in one arm, the arrival time delay between the two beams can then be controlled. Due to the energy conservation, the frequency sum of *s* and *i* lights is narrow, but the frequency difference can be broad. The widths of frequency sum and difference between the light in the two arms can be modulated by the length of the nonlinear crystal. The two beams are detected individually, so the frequency detection in one arm does not affect the time detection in the other. Since both beams are detected individually, the spectral and temporal resolutions can be controlled independently,^15,68,69^ This provides the theoretical basis for ultrafast time–frequency resolved spectroscopy.

### Model Hamiltonian and Ultrafast Dynamics Simulations

The model Hamiltonian for aggregated molecules in dilute solvents contains the electronic states and linear vibronic coupling, which are of the form:

7 $${Ĥ}_{M}=\underset{i,j}{\sum }\epsilon_{ij}\left|\Phi_{i}\right\rangle \left\langle \Phi_{j}\right|+\underset{i,j}{\sum }\underset{s}{\sum }g_{ij}^{s}Q_{s}\left|\Phi_{i}\right\rangle \left\langle \Phi_{j}\right|+\underset{s}{\sum }\frac{\omega_{s}}{2}\left(P_{s}^{2}+Q_{s}^{2}\right)$$

where |Φ_*i*_⟩ represents the *i*th diabatic electronic state. {ϵ_*ij*_} form a matrix of electronic Hamiltonian whose elements give the energies (diagonal) and the electronic couplings (off-diagonal) between the states. $P_{s}=-i\partial_{Q_{s}}$ and *Q*_*s*_ are the respective momentum and coordinate operators of the *s*th vibrational mode; ω_*s*_ denotes the frequency. $g_{ij}^{s}$ is the linear vibronic coupling strength. All parameters above are obtained from *ab initio* calculations.^46^ MRCI was carried out to calculate the electronic structures of the excited states with the one-particle basis functions prepared from time-dependent density functional theory^70^ (DFT/MRCI). The first two lowest-lying eigenstates with considerably large transition dipole moments from S_0_ are labeled as S_1_ and S_2_, having eigenenergies of 2.13 and 2.74 eV, respectively. The diagonal vibronic coupling $g_{ii}^{s}$ can be estimated by projecting the energy gradients onto the orientation space of normal modes. Only a single vibrational mode was considered to exhibit vibronic coupling, with its coupling strength fixed at $g_{ij}^{s}=800{\mathrm{cm}}^{-1}$ .^71^ The intermolecular exciton transfer is dominated by the electronic couplings arising from dipole–dipole interactions. Here, −500 cm^–1^ and −150 cm^–1^ are adopted for S_1_ and S_2_ transfer, respectively.^48^ Other parameters can be found in SI.

We carry out the TD-DMRG ultrafast dynamics simulations as an essential component of simulating the tr-QGS. According to previous work,^48,71,72^ we assume that the initial state is created by acting with the dipole operator on the ground state, namely, $\left|\psi \left(0\right)\right\rangle =V^{†}\left|\Psi_{0}^{\left(0\right)}\right\rangle$ that is further extrapolated by the *s*-photon bandwidth. Instead of solving the Schrödinger’s equation in a brutal-force manner, the TD-DMRG minimizes the Dirac–Frenkel functional ∥(i*∂*_*t*_ - *Ĥ*_*M*_)|ψ⟩∥^2^.^30^ This enables us to calculate the time-dependent wave functions |ψ­(*t*)⟩ and other observables in Figure 2, which further yield the tr-QGS in eq 5.

### Photon-Coincidence Counting

The coincidence counting signal from Figure 1c reads

8 $$S\left(\omega ,T\right)\propto \int_{-\infty }^{\infty }\left\langle a_{s}^{†}\left(\omega \right)a_{i}^{†}\left(t\right)a_{i}\left(t\right)a_{s}\left(\omega \right)\right\rangle dt$$

where $a_{i}\left(t\right)=\frac{E_{i}}{2\pi }\underset{v}{\sum }a_{i}\left(v\right)e^{-ivt}$ . *a*_*s*_(ω) and *a*_*i*_(ω) are the annihilation operators for the photons in *s* and *i* arms, respectively; the commutators are $\left[a_{s/i}\left(\omega \right),a_{s/i}^{†}\left(\omega^{\prime }\right)\right]=\delta_{\omega ,\omega^{\prime }}$ and $\left[a_{s}\left(\omega \right),a_{i}^{†}\left(\omega^{\prime }\right)\right]=0$ . In the QGS scheme, the fluorescence photons emitted from molecules go back into *s* arm again, and can thus be described by the optical field for *s* photons, i.e., $E_{s}\left(t\right)=\underset{\omega }{\sum }E_{s}a_{s}\left(\omega \right)e^{-i\omega t}$ . The molecules interact with the *s* photons, yielding the dipolar coupling *H*(*t*) = −V^†^(*t*) E_*s*_(*t*)—h.c.. Using the Dyson’s equation for *H*(*t*), one can carry out the perturbative expansion for eq 9 and obtain the time-resolved signal

$$\begin{array}{c} S\left(\omega ,T\right)\propto \int_{-\infty }^{\infty }\left[dt\right]\theta \left(t_{4}-t_{2}\right)\theta \left(t_{3}-t_{1}\right)\chi \left(t_{4},t_{3},t_{2},t_{1}\right)\\ C(\omega ;t,t_{4},t_{3},t_{2},t_{1}) \end{array}$$ 9

as given in eq 3. The four-point Green’s function of molecules χ­(*t*_4_, *t*_3_, *t*_2_, *t*_1_) = ⟨Ψ_0_|V­(*t*_1_)­V^†^(*t*_3_)­V­(*t*_4_)­V^†^(*t*_2_)|Ψ_0_⟩ gives the molecular response function, and the six-point Green’s function of the optical fields with the entangled-photons wave function is

10 $$\begin{array}{ll} C\left(\omega ;t,t_{4},t_{3},t_{2},t_{1}\right) & =e^{i\omega t_{4}}\left\langle \Phi \right|E_{s}^{†}\left(t_{1}\right)E_{s}\left(t_{3}\right)E_{s}^{†}\left(\omega \right)E_{i}^{†}\left(t\right)E_{i}\left(t\right)E_{s}\left(t_{2}\right)\left|\Phi \right\rangle \\ & \propto e^{i\omega (t_{4}-t_{3})}f\left(t_{2}-T_{s},t-T_{i}\right)f^{*}\left(t_{1}-T_{s},t-T_{i}\right) \end{array}$$

where $f\left(t_{2}-T_{s},t-T_{i}\right)=F\left[\Phi \left(\omega_{s},\omega_{i}\right)\right]$ is the dual Fourier transform of the wave function in eq 4. Thus, the spectral function $M\left[\left(\omega_{s}-\omega_{i}-\omega_{-}\right)\frac{\tau }{2}\right]=\exp\left[-{\left(\omega_{s}-\omega_{i}-\omega_{-}\right)}^{2}\tau^{2}/4\right]$ whose Fourier transform is responsible for the time-gating function $M\left(\frac{T-t}{\tau }\right)$ in eq 5. The frequency scale of the spectrum is from the term $e^{i\omega (t_{4}-t_{3})}$ interrogated with the four-point Green’s function ⟨Ψ_0_|V­(*t*_1_)­V^†^(*t*_3_)­V­(*t*_4_)­V^†^(*t*_2_)|Ψ_0_⟩. Detailed derivations are given in the SI.

## Supplementary Material



## Notes

Parameters in the Hamiltonian for the PBI-1 trimer used in computations, and some setups for TD-DMRG dynamics; derivation of Fisher information on time and frequency resolution; calculation of signal-to-noise ratio as a function of photon number; derivation of time-resolved quantum ghost spectroscopy in eq 5; derivation of the laser pump–probe spectra; derivation of the time-gated fluorescence spectrum; experimental feasibility and testable predictions; figure of tr-QGS with σ^–1^ = 1 ns (PDF)
