# Supplementary material for: Quantum Ghost Spectroscopy Reveals Hidden Coherence in Molecular Aggregates
Source: J Am Chem Soc. 2026 Oct 26;148(43):46648–57. doi: 10.1021/jacs.6c10272 (PMC13741849; doi:10.1021/jacs.6c10272)
Supplement: Supplementary file 1 [file ja6c10272_si_001.pdf]

Quantum Ghost Spectroscopy Reveals Hidden Coherence in Molecular Aggregates - Supporting Information.

Mingran Zhang[1], Yihe Xu[1], Vladislav V. Yakovlev[2,3,*], and Zhedong Zhang[1,4,*]

[1]Department of Physics, City University of Hong Kong, Kowloon, Hong Kong SAR
[2]MOE Key Laboratory for Biomedical Photonics, Wuhan National Laboratory for Optoelectronics, Huazhong University of Science and Technology, Wuhan, Hubei 430074, China
[3]Department of Biomedical Engineering, Texas A&M University, College Station, Texas 77843, United States
[4]Shenzhen Research Institute, City University of Hong Kong, Shenzhen, 518060, China

*Email: vladislav.yakovlev.tamu@gmail.com, zzhan26@cityu.edu.hk

Parameters of the PBI trimer and some TD-DMRG details

Table 1: All useful coefficients / constants in the maintext . All units in eV (dipole moment in ea0).

Coefficient	Value	Note
$\epsilon_{S_1^{(m)},S_1^{(m)}}$	2.13	excited energy of local S_1
$\epsilon_{S_2^{(m)},S_2^{(m)}}$	2.74	excited energy of local S_2
$\epsilon_{S_1^{(m)},S_1^{(n)}}$	-0.0619	electronic coupling between S_1 states. $\|m-n\|=1$
$\epsilon_{S_2^{(m)},S_2^{(n)}}$	-0.0186	electronic coupling between S_2 states. $\|m-n\|=1$
$\omega_1^{(m)}$	0.0368	frequency of vibrational modes
$\omega_2^{(m)}$	0.176	
$\omega_3^{(m)}$	0.0496	
$\omega_4^{(m)}$	0.168	
$\omega_5^{(m)}$	0.124	
$g^1_{S_1,S_1}$	0.0439	el-vib coupling g_{ij}^K
$g^2_{S_1,S_1}$	0.165	
$g^3_{S_2,S_2}$	0.0491	
$g^4_{S_2,S_2}$	0.163	
$g^5_{S_1,S_2}$	0.0992	
V_{S_0,S_1}	3.34	$S_0 \to S_1$ transition dipole
V_{S_0,S_2}	1.8	$S_0 \to S_2$ transition dipole

The used Hamiltonian parameters and dipole moments are shown in table 1. For TD-DMRG simulations, there are 6 excited states and 15 vibrational modes. They are put in the following order:

$$\{S_1^1, S_2^1, Q_1^1, Q_1^2, Q_3^1, Q_4^1, Q_5^1, S_2^2, S_2^1, Q_1^2, Q_2^2, Q_3^2, Q_4^2, Q_5^2, S_1^3, S_2^3, Q_1^3, Q_2^3, Q_3^3, Q_4^3, Q_5^3\} \tag{1}$$

to make one-dimensional lattice. The upper scripts represent the label of PBI-1 monomer. The number of reserved states during time evolution is set to 32 and the maximal occupational numbers of vibrational modes are all 16.

With the one-dimensional lattice above, an matrix product operator (MPS) decomposes the high-dimensional many-body coefficient tensor $c_{\sigma_1,\sigma_2,...,\sigma_n}$ of the quantum state $|\psi\rangle$, represented in the local Hartree-product basis $\{|\sigma_1\sigma_2\cdots\sigma_n\rangle\}$, as

$$|\psi\rangle = \sum_{\{\sigma_i=1\}}^{\{d_i\}} [M_1]^{\sigma_1} [M_2]^{\sigma_2} \cdots [M_n]^{\sigma_n} |\sigma_1\sigma_2\cdots\sigma_n\rangle. \tag{2}$$

Here, σ_i labels the local basis state of site i and represents a physical degree of freedom. In the present work, it may correspond to a cavity, vibrational mode, or excitonic state. The complete basis $\{|\sigma_1\sigma_2\cdots\sigma_n\rangle\}$ is constructed as the tensor product of all local basis states. The tensor $M_i^{\sigma_i}$ is associated with site i and carries one physical index σ_i. The dimensions of the physical and bond indices determine the size of the MPS representation. This decomposition is also commonly referred to as the tensor-train (TT) format.

Similar to MPS, $\hat{H}$ can be represented in matrix-product form as a matrix product operator (MPO),

$$\hat{H} = \sum_{\{\sigma_i\},\{\sigma_i'\}} [W_1]^{\sigma_1,\sigma_1'} [W_2]^{\sigma_2,\sigma_2'} \cdots [W_n]^{\sigma_n,\sigma_n'} |\sigma_1\sigma_2\cdots\sigma_n\rangle\langle\sigma_1'\sigma_2'\cdots\sigma_n'|. \tag{3}$$

Here, $W_i^{\sigma_i,\sigma_i'}$ is the local MPO tensor containing both bra and ket physical indices. For a given Hamiltonian, several strategies can be employed to construct its MPO representation. The MPS/MPO formalism adopts a one-dimensional chain topology and is particularly efficient for one-dimensional and quasi-one-dimensional systems with short-range interactions, where only moderate bond dimensions are typically required.

In general, the short-time evolution of a quantum state over a time step Δt is described by the propagation operator $\hat{U}(t, t+\Delta t)$,

$$|\psi(t+\Delta t)\rangle = \hat{U}(t, t+\Delta t)|\psi(t)\rangle \approx e^{-i\hat{H}(t)\Delta t/\hbar}|\psi(t)\rangle. \tag{4}$$

For a time-independent Hamiltonian, the time-evolution operator reduces to $\hat{U}(\Delta t) = \exp\left(-i\hat{H}_0\Delta t/\hbar\right)$. Within the MPS/MPO framework, a variety of time-propagation strategies have been developed. A particularly successful alternative is the time-dependent variational principle (TDVP), which projects the time-dependent Schrödinger equation onto the tangent space of the MPS manifold associated with the current state. The corresponding tangent-space projector can be written as

$$\hat{P}_{T,|\psi\rangle} = \sum_{i=1}^{n} \hat{P}^{L}_{i-1} \otimes \hat{I}_i \otimes \hat{P}^{R}_{i+1} - \sum_{i=1}^{n-1} \hat{P}^{L}_{i} \otimes \hat{P}^{R}_{i+1} = \sum_{i=1}^{n} \hat{P}^{(1)}_i - \sum_{i=1}^{n-1} \hat{P}^{(0)}_i, \tag{5}$$

where the left and right block projectors are defined as

$$\hat{P}^{L}_{i} = \sum_{\alpha_i} |\mathcal{L}^{[1:i]}_{\alpha_i}\rangle\langle\mathcal{L}^{[1:i]}_{\alpha_i}|, \qquad \hat{P}^{R}_{i} = \sum_{\alpha_i} |\mathcal{R}^{[i:n]}_{\alpha_i}\rangle\langle\mathcal{R}^{[i:n]}_{\alpha_i}|. \tag{6}$$

Here, $\mathcal{L}^{[1:i]}_{\alpha_i}$ and $\mathcal{R}^{[i:n]}_{\alpha_i}$ denote the α_i-th basis states of the left and right renormalized subspaces, respectively. The tangent space contains all infinitesimal variations that preserve the MPS structure at a fixed bond dimension. By projecting the time-dependent Schrödinger equation onto this tangent space, the time evolution remains confined to the variationally optimal MPS manifold. The resulting projected equation of motion is

$$i\hbar\frac{\partial}{\partial t}|\psi(t)\rangle = \hat{P}_{T,|\psi\rangle}\hat{H}_0|\psi(t)\rangle = \sum_{i=1}^{n}\hat{P}^{(1)}_i\hat{H}_0|\psi(t)\rangle - \sum_{i=1}^{n-1}\hat{P}^{(0)}_i\hat{H}_0|\psi(t)\rangle. \tag{7}$$

To enable adaptive control of the bond dimension during real-time propagation, the two-site TDVP algorithm evolves a two-site tensor,

$$i\hbar\frac{\partial}{\partial t}\left[M_{c,c+1}\right]^{\sigma_c,\sigma_{c+1}} = \sum_{\substack{\sigma'_c,\sigma'_{c+1},\\ \alpha'_{c-1},\alpha'_{c+1}}} \left[H^{\mathrm{eff},2}_{c,c+1}\right]^{\sigma_c,\sigma_{c+1},\sigma'_c,\sigma'_{c+1}} \left[M_{c,c+1}\right]^{\sigma'_c,\sigma'_{c+1}}. \tag{8}$$

The algorithm proceeds through a sequence of local two-site propagations followed by tensor decompositions and truncations. Unlike fixed-bond-dimension approaches, the two-site formulation allows the bond dimension to increase adaptively during time evolution, thereby reducing projection errors while introducing a controlled truncation error analogous to that encountered in conventional DMRG calculations. In practice, the two-site TDVP scheme generally provides improved accuracy and numerical robustness, albeit at a higher computational cost. Consequently, all simulations in this work are performed using the two-site TDVP approach.

Molecular model for the tr-QGS

Quantum-light interactions with molecules

For the tr-QGS scheme, the coincidence counting of photons is measured, yielding the signal

$$S(\omega, T) \propto \int_X \mathrm{d}t\, \langle\Psi(\infty)|\, a^\dagger_s(\omega)a^\dagger_i(t)a_i(t)a_s(\omega)\,|\Psi(\infty)\rangle \tag{9}$$

where $a_i(t) = \frac{\mathcal{E}_i}{2\pi}\sum_{\omega'} a_i(\omega')e^{-\mathrm{i}\omega' t}$. $a_s(\omega)$ and $a_i(\omega)$ are the annihilation operators for the photons in s and i arms, respectively; the commutators are $[a_{s/i}(\omega), a^\dagger_{s/i}(\omega')] = \delta_{\omega,\omega'}$, $[a_s(\omega), a^\dagger_i(\omega')] = 0$. The average $\langle\cdots\rangle$ in Eq.(9) is taken over the wave function of molecule and entangled light. Normally $X \sim 5$ ns arising from the instrumental response of single-photon detectors. The integral over t can be extended to ∞, when the dynamics during picoseconds is of the most interest.

The dipolar interaction between molecules and photons reads

$$H(t) = -\mathrm{V}^{+}(t)E_s(t) - \mathrm{V}^{-}(t)E_s^{\dagger}(t) \tag{10}$$

in the interactive picture where the rotating-wave approximation (RWA) was used; $\mathrm{V}^{+(-)}(t)$ is the raising (lowering) part of the electric dipole operator. $E_s(t)$ and $E_i(t)$ denote the respective electric fields (positive-frequency) in s and i arms, i.e.,

$$E_s(t) = \frac{1}{2\pi}\sum_{\omega}\mathcal{E}_{s,\omega}a_s(\omega)e^{-\mathrm{i}\omega t}, \quad E_i(t) = \frac{1}{2\pi}\sum_{\omega}\mathcal{E}_{i,\omega}a_i(\omega)e^{-\mathrm{i}\omega t}. \tag{11}$$

The entangled twin photons are generated from the nonlinear crystals via SPDC process—depicted in Fig.1(c) in main text—so that the s photons are incident to molecules whereas the i photons propagate freely.

The full wave function of the joint molecule + photon system follows the dynamics

$$|\Psi(t)\rangle = \mathrm{U}(t)\hat{\mathcal{T}}e^{-\mathrm{i}\int_{-\infty}^{t}H(t')\mathrm{d}t'}|\Psi_0\rangle\otimes|\Phi\rangle \tag{12}$$

with $\mathrm{U}(t)$ being the *Green's propagator* of molecules in absence of the optical fields. $|\Psi_0\rangle$ and $|\Phi\rangle$ are the initial states of molecules and photon fields, and they are assumed to be disentangled before the interactions. We further assume that the molecules are at electronic ground state before the action of light. The Dyson's equation for $H(t)$ enables the 2nd-order perturbation series for the wave function, yielding

$$|\Psi(\infty)\rangle = (-\mathrm{i})^2\iint_{-\infty}^{\infty}\mathrm{d}t_2\mathrm{d}t_1\theta(t_2-t_1)\mathrm{U}(\infty)H(t_2)H(t_1)|\Psi_0\rangle\otimes|\Phi\rangle\,. \tag{13}$$

Making use of Eqs.(9)-(13), the coincidence counting signal reads

$$S(\omega,T)\propto\int_{-\infty}^{\infty}[\mathrm{d}t]\theta(t_4-t_2)\theta(t_3-t_1)M(t_4,t_3,t_2,t_1)C(\omega;t,t_4,t_3,t_2,t_1), \tag{14a}$$

$$M(t_4,t_3,t_2,t_1) = \langle\Psi_0|\,\mathrm{V}^{-}(t_1)\mathrm{V}^{+}(t_3)\mathrm{V}^{-}(t_4)\mathrm{V}^{+}(t_2)\,|\Psi_0\rangle \tag{14b}$$

$$C(\omega;t,t_4,t_3,t_2,t_1) = e^{\mathrm{i}\omega t_4}\langle\Phi|\,E_s^{\dagger}(t_1)E_s(t_3)E_s^{\dagger}(\omega)E_i^{\dagger}(t)E_i(t)E_s(t_2)\,|\Phi\rangle \tag{14c}$$

where $[\mathrm{d}t]\equiv\mathrm{d}t\mathrm{d}t_4\mathrm{d}t_3\mathrm{d}t_2\mathrm{d}t_1$ is a bookkeeping notation.

Chemical dynamics of molecular aggregates

The dynamics of electronic excited states, e.g., in PBI-1 aggregates, is involved in the four-point Green's function in Eq.(14b). This casts into

$$M(t_4,t_3,t_2,t_1) = e^{-\mathrm{i}E_0(t_2-t_1)}\langle\Psi_0(q)|\,\mathrm{V}^{-}\mathrm{U}(t_1-t)\cdot\mathrm{U}(t-t_3)\mathrm{V}^{+}\mathrm{U}(t_3-t_4)\mathrm{V}^{-}\mathrm{U}(t_4-t)\cdot\mathrm{U}(t-t_2)\mathrm{V}^{+}\,|\Psi_0(q)\rangle \tag{15}$$

by noting the identity $\mathrm{U}(t_3-t_4)=\mathrm{U}(t_3-t)\mathrm{U}(t-t_4)$. The model Hamiltonian of aggregated molecules reads

$$\hat{H}_{\mathrm{el}} = \sum_i\varepsilon_i|\psi_i\rangle\langle\psi_i| + \sum_{i\neq j}V_{ij}|\psi_i\rangle\langle\psi_j| + \sum_{i,j}\sum_s g_{ij}^sQ_s|\psi_i\rangle\langle\psi_j| + \sum_s\frac{\omega_s}{2}\left(-\frac{\partial^2}{\partial Q_s^2}+Q_s^2\right) \tag{16}$$

in the diabatic basis $|\psi_i\rangle$, where the onsite energy ε_i, electric coupling V_{ij}, vibrational frequency ω_s and electron-vibration coupling g_{ij}^s can be obtained from quantum-chemistry calculations. $|\psi_i\rangle$ can be chosen as the adiabatic electronic state at the PBI-1 monomer, and Q_s is the coordinate of the sth normal-mode vibration. The electronic couplings V_{ij} are weak, i.e., $|V_{ij}| < |\varepsilon_i-\varepsilon_j|$, responsible for aggregating the monomers.

In PBI-1 trimers, there are 6 electronic excited states and 15 vibrational modes (S_1, S_2 and 5 vibrations are for a monomer) where 6 modes are of low-frequency, i.e., < 400 cm^{-1}. The solvent dephasing can be involved at phenomenological level, which gives extra line broadening. The quantum dynamics is governed by the molecular wave function $|\Psi(Q,t)\rangle$ which obeys the equation of motion

$$\left(\mathrm{i}\partial_t - \hat{H}_{\mathrm{el}}\right)|\Psi(Q,t)\rangle = 0. \tag{17}$$

Eq.(17) is elaborated by the TD-DMRG method, which solves for the Green's propagator

$$\mathrm{U}(t-t_0)=e^{-\mathrm{i}\hat{H}_{\mathrm{el}}(t-t_0)}. \tag{18}$$

Defining the operator

$$\mathrm{Y}(t_3-t,t_4-t)=\mathrm{V}^{+}(t_3-t)\mathrm{V}^{-}(t_4-t), \tag{19}$$

one recasts Eq.(15) into

$$M(t_4,t_3,t_2,t_1)=e^{-\mathrm{i}E_0(t_2-t_1)}\left\langle\Psi_0(q)\right|\mathrm{V}^{-}\mathrm{U}(t_1-t)\mathrm{Y}(t_3-t,t_4-t)\mathrm{U}(t-t_2)\mathrm{V}^{+}\left|\Psi_0(q)\right\rangle. \tag{20}$$

It turns out that $\mathrm{Y}(t_3-t,t_4-t)$ generates the spectral lines and governs their shapes. A sophisticated way of interpreting Eq.(20) is gained from a three-step process, i.e.,

1. Preparation of an initial excited state through a doorway action $\mathrm{V}^{+}|\Psi_0(q)\rangle$;
2. Propagation of excited-state wave packet during $t-t_2$;
3. Window action within t_4-t that de-excites the excited-state wave packet.

Green's function of optical fields

Now let us calculate the six-point Green's function of the light fields in Eq.(14c). Using the entangled twin photons with the wave function

$$|\Phi\rangle=\iint_{-\infty}^{\infty}\mathrm{d}\omega\mathrm{d}\omega'\phi(\omega,\omega')a_s^{\dagger}(\omega)a_i^{\dagger}(\omega')\left|0\right\rangle, \tag{21}$$

one has the two-photon amplitude

$$\langle 0|E_i(t)E_s(\tau)|\Phi\rangle=\mathcal{E}_s\mathcal{E}_i f(\tau-T_s,t-T_i) \tag{22}$$

where $f(\tau-T_s,t-T_i)=\mathcal{F}[\phi(\omega_s,\omega_i)]$. Using Eq.(22), it gives

$$\begin{aligned}C(\omega;t,t_4,t_3,t_2,t_1)&=e^{\mathrm{i}\omega t_4}\langle\Phi|E_s^{\dagger}(t_1)E_i^{\dagger}(t)|0\rangle\langle 0|E_s(t_3)E_s^{\dagger}(\omega)|0\rangle\langle 0|E_i(t)E_s(t_2)|\Phi\rangle\\&=(2\pi)^{-1}|\mathcal{E}_s|^4|\mathcal{E}_i|^2e^{\mathrm{i}\omega(t_4-t_3)}f(t_2-T_s,t-T_i)f^*(t_1-T_s,t-T_i).\end{aligned} \tag{23}$$

Notice that that the two-photon wave function is of a factorized form with

$$\phi(\omega_s,\omega_i)=\sqrt{\tau}A(\omega_s+\omega_i-\omega_+)M\Big[(\omega_s-\omega_i-\omega_-)\frac{\tau}{2}\Big] \tag{24}$$

Here $A(\omega_s+\omega_i-\omega_+)=\exp\left[-(\omega_s+\omega_i-\omega_+)^2\frac{1}{\sigma^2}\right]$ and $M\left[(\omega_s-\omega_i-\omega_-)\frac{\tau}{2}\right]=\exp\left[-(\omega_s-\omega_i-\omega_-)^2\frac{\tau^2}{4}\right]$ are two Gaussian functions. one can success from Eq.(22), obtaining

$$\begin{aligned}f(t_2-T_s,t-T_i)&=\frac{1}{(2\pi)^2}\iint_{-\infty}^{\infty}\mathrm{d}v_+\mathrm{d}v_-e^{-\mathrm{i}v_+(t_2+t-T_s-T_i)}e^{-\mathrm{i}v_-(t_2-t+T)/2}A(2v_+-\omega_+)M\left[(v_--\omega_-)\frac{\tau}{2}\right]\\&=\frac{1}{\tau}e^{-\mathrm{i}\omega_+(t_2+t-T_s-T_i)/2}e^{-\mathrm{i}\omega_-(t_2-t+T)/2}\mathcal{A}\left(\frac{t_2+t-T_s-T_i}{2}\right)\mathcal{M}\left(\frac{t_2-t+T}{\tau}\right)\end{aligned} \tag{25}$$

where $\mathcal{A}(t)=\mathcal{F}[A(\omega)]$ and $\mathcal{M}(t/\tau)=\mathcal{F}[M(\omega\tau)]$. Thus the six-point Green's function of the fields can be found, i.e.,

$$\begin{aligned}C(\omega;t,t_4,t_3,t_2,t_1)=&\frac{|\mathcal{E}_s|^4|\mathcal{E}_i|^2}{2\pi\tau}e^{\mathrm{i}\omega(t_4-t_3)}e^{-\mathrm{i}(\omega_++\omega_-)(t_2-t_1)/2}\mathcal{A}\left(\frac{t_2+t-T_s-T_i}{2}\right)\mathcal{M}\left(\frac{t_2-t+T}{\tau}\right)\\&\times\mathcal{A}^*\left(\frac{t_1+t-T_s-T_i}{2}\right)\mathcal{M}^*\left(\frac{t_1-t+T}{\tau}\right).\end{aligned} \tag{26}$$

Time-energy-resolved photon-coincidence counting

Using Eqs.(20) and (26), one recasts Eq.(14a) into

$$S(\omega,T) \propto \frac{1}{\tau}\int_{-\infty}^{\infty}[\mathrm{d}t]\theta(t_2-t_4)\theta(t_1-t_3)e^{\mathrm{i}[E_0(t_2-t_1)-\omega(t_4-t_3)]}e^{\mathrm{i}E_0(t_2-t_1)}e^{-\mathrm{i}\omega(t_4-t_3)}$$
$$\times \langle\Psi_0(q)|\,\mathrm{V}^-\mathrm{U}(-t_1)\mathrm{Y}(-t_3,-t_4)\mathrm{U}(t_2)\mathrm{V}^+\,|\Psi_0(q)\rangle$$
$$\times\,\mathcal{A}\left(\frac{2t-t_2-T_s-T_i}{2}\right)\mathcal{M}\left(\frac{T-t_2}{\tau}\right)\mathcal{A}^*\left(\frac{2t-t_1-T_s-T_i}{2}\right)\mathcal{M}^*\left(\frac{T-t_1}{\tau}\right). \tag{27}$$

Reordering the integrals

$$\int_{-\infty}^{\infty}\mathrm{d}t\int_{-\infty}^{\infty}\mathrm{d}t_4\int_{-\infty}^{\infty}\mathrm{d}t_3\int_{t_4}^{\infty}\mathrm{d}t_2\int_{t_3}^{\infty}\mathrm{d}t_1=\int_{-\infty}^{\infty}\mathrm{d}t_2\int_{-\infty}^{\infty}\mathrm{d}t_1\int_{-\infty}^{\infty}\mathrm{d}t\int_{-\infty}^{t_2}\mathrm{d}t_4\int_{-\infty}^{t_1}\mathrm{d}t_3 \tag{28}$$

one can carry out the integrals over t_4, t_3 such that

$$\int_{-\infty}^{t_2}\mathrm{d}t_4\int_{-\infty}^{t_1}\mathrm{d}t_3e^{-\mathrm{i}\omega(t_4-t_3)}\mathrm{Y}(-t_3,-t_4)\approx\mathrm{V}^+(-\omega)\mathrm{V}^-(\omega). \tag{29}$$

The integral over t results in

$$\int_{-\infty}^{\infty}\mathrm{d}t\mathcal{A}\left(\frac{2t-t_2-T_s-T_i}{2}\right)\mathcal{A}^*\left(\frac{2t-t_1-T_s-T_i}{2}\right)=W\left(\frac{t_2-t_1}{2}\right) \tag{30}$$

with the W function defined as $W(x)=\int_{-\infty}^{\infty}\mathcal{A}(z)\mathcal{A}^*(z+x)\mathrm{d}z$. On substituting Eqs.(29) and (30) into Eq.(27), one obtains the tr-QGS

$$S(\omega,T)\propto\frac{1}{\tau}\iint_{-\infty}^{\infty}\mathrm{d}t_2\mathrm{d}t_1e^{\mathrm{i}E_0(t_2-t_1)}W\left(\frac{t_2-t_1}{2}\right)\mathcal{M}\left(\frac{T-t_2}{\tau}\right)\mathcal{M}^*\left(\frac{T-t_1}{\tau}\right)$$
$$\times\langle\Psi_0(q)|\,\mathrm{V}^-\mathrm{U}(-t_1)\mathrm{V}^+(-\omega)\mathrm{V}^-(\omega)\mathrm{U}(t_2)\mathrm{V}^+\,|\Psi_0(q)\rangle\,. \tag{31}$$

Noting the fact $W^*(-x)=W(x)$ and $[\mathrm{V}^-(\omega)]^\dagger=\mathrm{V}^+(-\omega)$, we learn from Eq.(31) that $S(\omega,T)$ is a real function. Eq.(31) is the most general form for the tr-QGS.

As we are interested in the entangled photons that are spectrally anti-correlated, i.e., $\sigma\ll\tau^{-1}$, the function $\mathcal{A}(t)$ is substantially broader than $\mathcal{M}(t/\tau)$ in time domain. The temporal integrals in Eq.(31) are effective within a short window, during which the function $W(\frac{t_2-t_1}{2})$ does not vary significantly. Therefore the *slowly-varying approximation* $W(x)\approx W(0)$ is allowed. Using the Dirac's completeness relation, one has

$$\langle\Psi_0(q)|\,\mathrm{V}^-\mathrm{U}(-t_1)\mathrm{V}^+(-\omega)\mathrm{V}^-(\omega)\mathrm{U}(t_2)\mathrm{V}^+\,|\Psi_0(q)\rangle=\sum_{q'}\langle\Psi_0(q)|\,\mathrm{V}^-\mathrm{U}(-t_1)\mathrm{V}^+(-\omega)\,|\Psi_0(q')\rangle$$
$$\times\langle\Psi_0(q')|\,\mathrm{V}^-(\omega)\mathrm{U}(t_2)\mathrm{V}^+\,|\Psi_0(q)\rangle\,. \tag{32}$$

Inserting Eq.(32) into Eq.(31), the tr-QGS is of the form

$$S(\omega,T)\propto\frac{1}{\tau}\sum_{q'}\left|\int_{-\infty}^{\infty}\mathrm{d}te^{\mathrm{i}E_0t}\langle\Psi_0(q')|\,\mathrm{V}^-(\omega)\mathrm{U}(t)\mathrm{V}^+\,|\Psi_0(q)\rangle\,\mathcal{M}\left(\frac{T-t}{\tau}\right)\right|^2 \tag{33}$$

which reveals Eq.(5) in main text.

Fisher information for tr-QGS

Fisher information (FI) is a fundamental quantity in theoretical metrology and quantum sensing, characterizing the minimum uncertainty in estimating an unknown parameter. The Cramér-Rao bound provides a lower bound on the estimation resolution, for example, for the time delay T [1–4]

$$(\Delta T)^2 \geqslant \frac{1}{mF(T)} \tag{34}$$

where m is the repeated number of experiments and $F(T)$ is the Fisher information of time delay T. The Cramér-Rao bound sets a lower limit on the uncertainty with which a parameter can be estimated. For a single parameter, the Fisher information is obtained from the second moment of the derivative of the log-likelihood function with respect to the parameter.: [1]

$$F(x) = \int p(\xi|x)\left(\frac{\partial \ln p(\xi|x)}{\partial x}\right)^2 \mathrm{d}\xi \tag{35}$$

where ξ is the measurement result and x is the parameter. In our scheme, ξ is the coincide counting rate. $p(\xi|x)$ is the conditional probability. Multiple parameters $\boldsymbol{x} = \{x_1, x_2, \cdots, x_n\}$ requires an $n \times n$ Fisher information matrix for evaluating the variance, which gives: [5]

$$F_{ij}(\boldsymbol{x}) = \int p(\xi|\boldsymbol{x}) \frac{\partial \ln p(\xi|\boldsymbol{x})}{\partial x_i} \frac{\partial \ln p(\xi|\boldsymbol{x})}{\partial x_j} \mathrm{d}\xi \tag{36}$$

As a brief example, a Gaussian beam in the time domain represents $G(t) = \exp\big((t-T)^2/2\sigma^2\big)$, for T is the central time of the beam and σ is its standard deviation. The FI of the measured parameter T reads $F(T) = 1/\sigma^2$. Thus, the minimal uncertainty of the central time is bounded by the Cramér-Rao bound as

$$(\Delta T)^2 \geqslant \sigma^2 \tag{37}$$

which means the minimum distance required to resolve two such peaks is $\Delta T = \sigma$.

The time- and frequency-resolved tr-QGS signal is not a standard probability density function. In the limits $\tau \to 0$ and $\gamma \to 0$, the signal broadening vanishes, yielding full time and frequency resolution. Under this condition, the signal at any point (ω_0, T_0) would not be affected by contributions from neighboring points. The signal can be thus written as

$$S(\omega_0; T_0) = \sum_{\nu_1 \nu_2 \cdots} \frac{1}{\tau} \iint \mathrm{d}\omega \mathrm{d}T |S_0(\omega, T)|^2 \delta(\omega - \omega_0)\delta(T - T_0). \tag{38}$$

Here $S_0(\omega, T) = \langle \Psi_0^{(\nu_1, \nu_2, \cdots)} | \mathrm{V}_0(\omega) U(t) \mathrm{V}^\dagger | \Psi_0^{(0,0,\cdots)} \rangle$ is the signal without broadening, $\delta(\omega)$ is the Dirac delta function. Here $\mathrm{V}_0(\omega) = \int dt' e^{i\omega t'} \mathrm{V}(t')$ is the pure spectrum ignoring decay. The frequency line width can be thus written as

$$\mathrm{V}(\omega) = \int dt' e^{i\omega t' - \gamma |t'|} \mathrm{V}(t') = \frac{2\gamma}{\gamma^2 + \omega^2} * \mathrm{V}_0(\omega). \tag{39}$$

The time gated function $\mathcal{M}(\frac{T-t}{\tau})$ can be written in the similar form to achieve

$$S(\omega; T) = \tau \sum_{\nu_1 \nu_2 \cdots} \left| \int_{-\infty}^{\infty} \mathrm{d}t S_0(\omega, T) \frac{1}{\tau} \mathcal{M}(\frac{T-t}{\tau}) \right|^2 = \sum_{\nu_1 \nu_2 \cdots} |S_0 * G(T;\tau)|^2 \tag{40}$$

Here $G(T;\tau) = \frac{1}{\sqrt{\pi}\tau} \mathcal{M}(T;\tau) = \frac{1}{\sqrt{\pi}\tau} \exp\big(-T^2/\tau^2\big)$ is the normalized probability density function of Gaussian function $\mathcal{M}$. By applying the above equations all together, we can get

$$S(\omega_0; T_0) \propto \sum_{\nu_1 \nu_2 \cdots} \iint \mathrm{d}\omega \mathrm{d}T |S_0(\omega, T) \cdot \left(\delta(\omega - \omega_0) * \frac{2\gamma}{\gamma^2 + \omega^2} \right) \cdot \left(\delta(T - T_0) * G(T;\tau) \right) |^2. \tag{41}$$

It is clearly shown in eq. (41) that the frequency broadening is described by the Lorentzian function $2\gamma/(\gamma^2 + \omega^2)$, and the temporal line width corresponds to the Gaussian distribution $G(T;\tau)$. The line widths of frequency and time are decided by γ and τ separately, which lead to the independent control of both resolutions as shown in Figure 4 and 5 in the main text.

Thus, the classical Fisher information matrix(CFIM) of the signal based on time T and frequency γ_0 reads

$$F = \begin{pmatrix} \frac{1}{2\gamma^2} & 0 \\ 0 & \frac{2}{\tau^2} \end{pmatrix} \tag{42}$$

The product of standard deviation related to time and frequency is $\Delta T \Delta\omega = \gamma\tau$. By inserting the simulation data in the main text, the lowest joint resolution is $\Delta T\Delta\omega = 0.11$ with $\tau = 7$ fs and $\gamma^{-1} = 10$ fs. For the product of standard deviation as a function of τ, the results are 0.80 and 3.18 at $\tau = 50$ fs and $\tau = 200$ fs. By picking $\gamma^{-1} = 5$ fs and 2 fs, the products are 0.22 and 1.11. Although large time window and low spectral resolution of the detector would compromise the joint resolution capability, as demonstrated by the cases of $\tau = 200$ and $\gamma^{-1} = 2$ fs, which fall within the resolution of classical time-resolved signal regime, we find that all the deviation products are lower than the time gated signal in eq. (51), as illustrated in Figure 1(e).

Signal-to-noise ratio of tr-QGS versus photon number

Generally, the ideal entangled photon pairs promise exact number of photons in both beams, which causes the variance of photon number vanishes. This unique property for quantum entanglement provides a pure signal without noise, e.g., for a Fock state $|n\rangle$, the variance of photon number is $(\Delta n_{\text{fock}})^2 = 0$. As a comparison, a coherent state $|\alpha\rangle$ ($|\alpha|^2 = n$) with lowest uncertainty would cause a photon number variance as $(\Delta n_{\text{coh}})^2 = n$. However, in real experiments, we need to consider the impact of photon losses, which removing the divergence of signal-to-noise ratio (SNR) of entangled photon pairs, as shown in Figure 1.

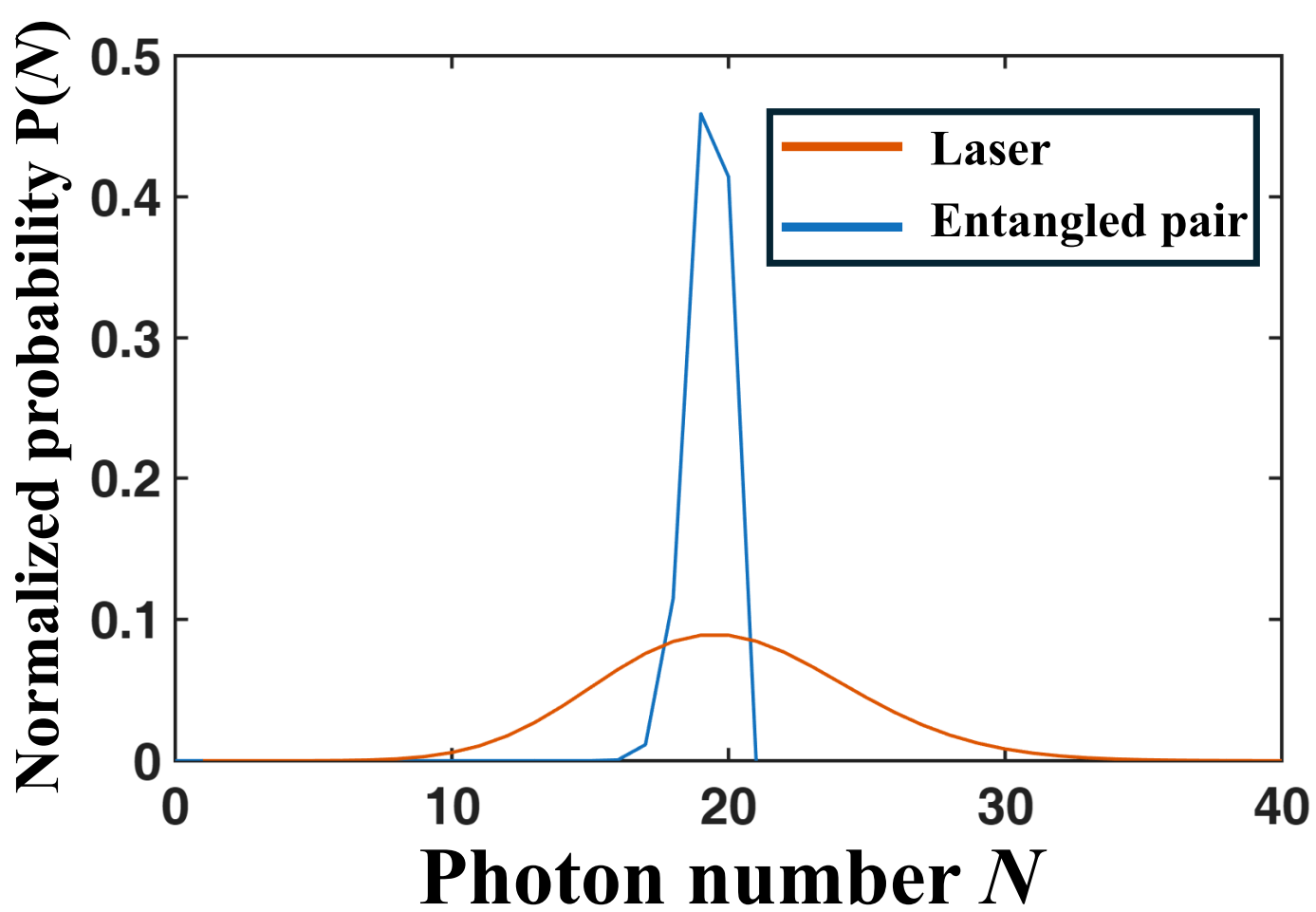

Figure 1: Probability distribution of entangled photon pair versus laser. The entangled photon pair shows a a higher concentration distribution, namely sub-Poisson distribution to enhance the SNR. Mean photon number of coherent state is $\bar{n} = 20$, the number of entangled photon pairs (before loss) is 20 for direct comparison. Photon detection efficiency is $p = 0.95$.

Photon number resolved detector

The photon number resolved detector has the ability to figure out different number of photons reaching the detector at the same time, with a small probability γ to record less photon due to the quantum efficiency

and photon number resolution.[6] In our model, $\gamma = 0.05$ is small enough to ignore events with more than one photon loss. The mean photon number recorded would be $E(N) = N - \gamma$ with N the number of generated pairs of photons. The variance thus would be

$$(\Delta N_{\text{loss}})^2 = \gamma + 3\gamma^2 \tag{43}$$

with the corresponding SNR

$$\text{SNR} = \frac{E(N)}{\text{Var}(N)} = \frac{N - \gamma}{\sqrt{\gamma + 3\gamma^2}} \approx N \tag{44}$$

The SNR of N entangled photon pairs still maintains linear level under the assumption of photon loss number resolved detector, overcoming the SNL of laser-based coherent state.

Joint detection with photon loss

The single photon detector array can also work as a pseudo number resolved detector. In this case, one photon in both beams arriving the detectors at the same time would be recorded as a click. Thus, for N entangled photon pairs, only when the number of photons in s and i beams $n_s = n_i = k$, the detector would record k clicks. The quantum efficiency decides the probability that a photon can be detected by the single photon detector, e.g. $p = 0.95$ as a commonly used quantum efficiency.[7] In this scenario, the probability of detecting k photons in one beam follows binomial distribution p_k, while the joint detection follows the distribution $\frac{p_k^2}{A}$ with the renormalization factor A, where $A = \sum_k p_k^2$.

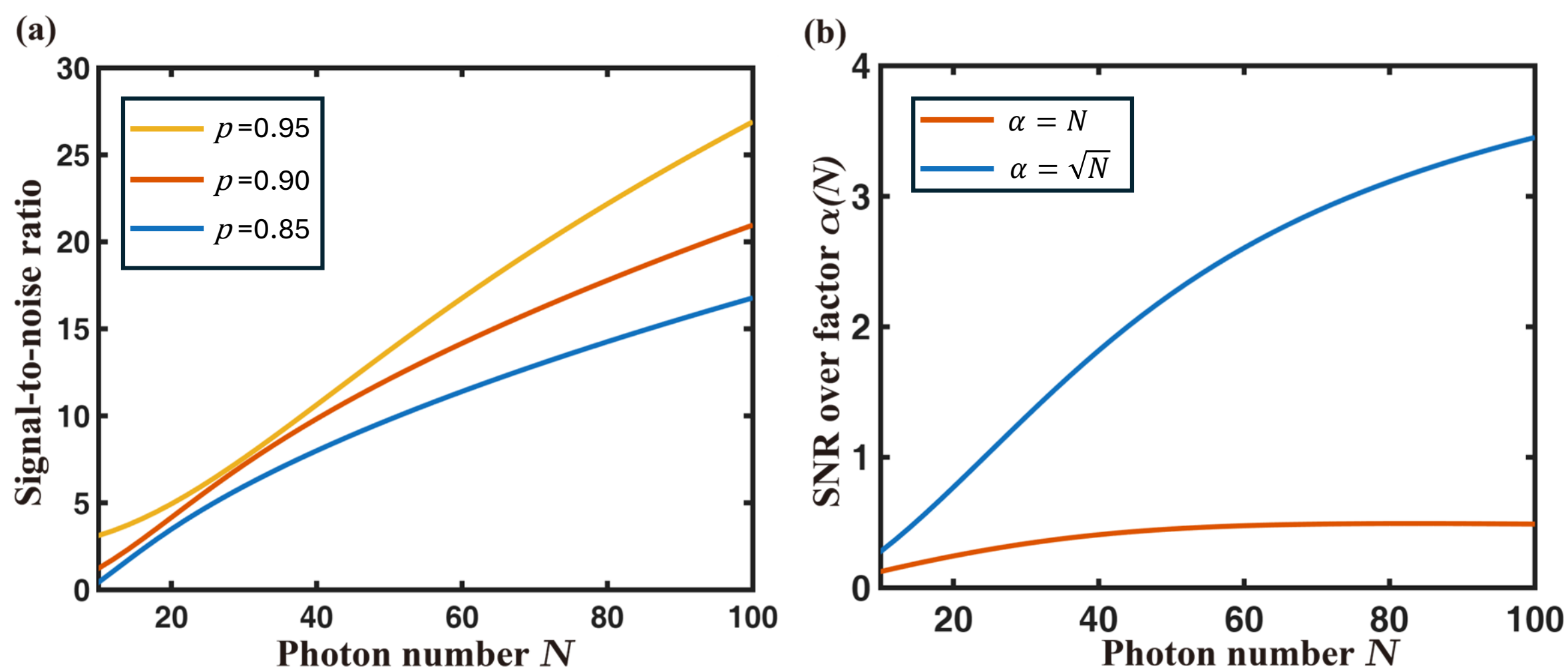

Figure 2: Simulation of entangled light SNR as a function of photon number N. (a) SNR of lossy joint detection with different detection efficiency p. (b) SNR of $p = 0.95$ compare to linear and sublinear extensions.

Figure 2a shows the simulated results of SNR. The SNR shows a near linear dependence of N. The gradient of the scaling between SNR and photon number would slightly decrease for detectors with lower quantum efficiency, but maintains a nearly linear dependence. To make a clear comparison, 2b represents the behavior of $\text{SNR}/\sqrt{N}$ and SNR/N. It shows that the curve of SNR/N trends to be a constant at 0.5, meaning a linear relationship between $2N$ photons and SNR. Since N entangled photon pairs consist $2N$ photons to represent N coincide counting events, the SNR of entangled photon pairs includes a factor 0.5. Both noise analysis of detectors supports our SNR discussion associates to Figure 1d in the main text. If the pump laser intensity is increased largely before injection into the nonlinear crystal, the spontaneous parametric down-conversion process generates squeezed light rather than an entangled photon pair, which is a scenario that requires further investigation. Notably, squeezed light has also been shown to exhibit a linear signal-to-noise ratio (SNR) in recent theoretical spectroscopy studies.[4,8]

Time-resolved fluorescence

We define the time and frequency gate functions as follows:

$$F_t(\sigma_T; T, t') = e^{-\frac{1}{2}\sigma_T^2 (t'-T)^2}, \tag{45}$$

$$F_\omega(\sigma_\omega; \omega, \omega') = e^{-\frac{1}{4\sigma_\omega^2}(\omega'-\omega)^2}. \tag{46}$$

The gated fluorescence signal is the photon numbers detected, thus represents as

$$S(T,\omega) = n_{T,\omega} = \int \mathrm{d}t'' \left\langle E^\dagger_{t,\omega}(t'') E_{t,\omega}(t'') \right\rangle. \tag{47}$$

Here the input laser source is a set of coherent state

$$|\phi_{\text{laser}}\rangle = \int \mathrm{d}\nu e^{\frac{-(\nu-\nu_0)^2}{2\Omega^2}} |\alpha(\nu)\rangle, \tag{48}$$

where $|\alpha(\nu)\rangle$ is the coherent state at frequency ν, ν_0 is the central frequency of the laser.

The electric field operator $E_{t,\omega}(t'')$ contains the information of time and frequency gates:

$$E_{t,\omega}(t'') = \int \mathrm{d}t' F_\omega(t''-t', \omega) F_t(t', T) E(t'). \tag{49}$$

Here $F_\omega(t''-t', \omega)$ is the Fourier transform of Equation 46.

Following the same perturbation method of entangled signal, we achieve the representation of 47 as:

$$S(t,\omega) \propto \mathrm{Re}\frac{1}{\tau}\sum_{\nu_1\nu_2\cdots}\int \mathrm{d}t' \int \mathrm{d}\omega' \left| \int_{-\infty}^{\infty} \mathrm{d}t e^{iE_0 t} \langle \Psi_0^{(\nu_1,\nu_2,\cdots)} | \mathrm{V}(\omega')\mathrm{U}(t)\mathrm{V}^\dagger | \Psi_0^{(0,0,\cdots)} \rangle e^{-\frac{\Omega^2}{4}(t-t')^2} \right|^2 W(T,\omega; t', \omega'). \tag{50}$$

Here $W(T,\omega;t',\omega')$ is the total time- and frequency-gated function

$$W(T,\omega;t',\omega') = e^{-\frac{1}{2\tilde{\sigma}_T^2}(t'-T)^2} e^{-\frac{1}{2\tilde{\sigma}_\omega^2}(\omega'-\omega)^2} e^{-i\theta(\omega'-\omega)(t'-T)}. \tag{51}$$

The total resolution $\tilde{\sigma}_T^2 = \left(\frac{1}{\sigma_T^2} + \frac{1}{\sigma_\omega^2}\right)$, $\tilde{\sigma}_\omega^2 = \sigma_T^2 + \sigma_\omega^2$, maintains the Fourier limit. $\theta = \frac{\sigma_T^2}{\sigma_T^2 + \sigma_\omega^2}$ is an extra phase term associated with ω and t.

Through Equation 51, the temporal resolution becomes high when both gates are broad, which would cause the low frequency resolution and vice versa. To reach the lower bound of the joint resolution, both gates must be set to 1 fs^{-1}. In this regime, however, the achievable time and frequency resolution fails to meet the experimental requirements. Moreover, unlike the scenario in eq. (42), the time-gated fluorescence signal cannot provide the same level of resolution as obtained with entangled photons.

Laser pump-probe spectroscopy

The total Hamiltonian with external field for pump probe spectroscopy reads:

$$\begin{aligned} \hat{H}(t) &= \hat{H}_0 + \hat{H}'(t) \\ \hat{H}'(t) &= \hat{V}[\mathcal{E}_1(t) + \mathcal{E}_2(t)] + h.c., \end{aligned} \tag{52}$$

where the envelop of light field is $\mathcal{E}_n(t) = \exp\left[-\frac{(t-t_{c,n})^2}{\sigma^2} + i\omega_c(t - t_{c,n})\right]$. We use three incident Gaussian pulses together with a probe pulse for measurement. The parameter $t_{c,n}$ denotes the central time of the n-th pulse. The time delay between adjacent pulses are defined as T. The initial state is set to a direct product of molecular ground state and vaccum photon state, $|\psi(0)\rangle = |\Psi_g^{0\cdots 0}\rangle \otimes |0\rangle$. The time-dependent Schrödinger's equation is written as:

$$|\frac{d\psi}{dt}\rangle = -i\left[\hat{H}_0 + \hat{H}'(t)\right]|\psi\rangle. \tag{53}$$

The two-dimensional electronic spectroscopy (2DES) signal is related to the third-order density operator, where the nonlinear polarization and corresponding rephasing 2DES signal is written as:

$$S(T_1,\Omega) \propto \mathcal{R}\int_{-\infty}^{\infty} dT_2 e^{+i\Omega T_2}\hat{P}^{(3)}(T_2,T_1), \tag{54}$$

with

$$\hat{P}^{(3)}(T_2,T_1) = \langle \hat{V}\rho^{(3)}\rangle = (-i)^3\int[\mathrm{d}t_1,t_2,t_3]\,\langle\psi(0)|\,\hat{H}'(t_1)U^\dagger(t_2-t_1)\hat{H}'(t_2)U^\dagger(t-t_2)\hat{V}U(t-t_3)\hat{H}'(t_3)\,|\psi(0)\rangle\,. \tag{55}$$

and relationships:

$$\begin{aligned}\tau_1 = t_2 - t_1 \quad \tau_2 = t_3 - t_2 \quad \tau_3 = t - t_3\\ T_1 = t_{c,2} - t_{c,1} \quad T_2 = t - t_{c,2}\end{aligned} \tag{56}$$

where $t_{c,i}$ is the central time of the i-th pulse. Within these conditions, we can then extract the rephasing GSB and SE signals respectively.

$$\begin{aligned}S_{\mathrm{GSB,R}} = \mathcal{R}\int_{-\infty}^{\infty} dT_2 e^{i\Omega T_2}\iiint_0^\infty d[\tau_3,\tau_2,\tau_1]\,\langle\Psi_g^{(0)}|\,\hat{V}U^\dagger(\tau_1)\hat{V}^\dagger U^\dagger(\tau_2+\tau_3)\hat{V}U(\tau_3)\hat{V}^\dagger\,|\Psi_g^{(0)}\rangle\\ \times\mathcal{G}(T_2-\tau_3)\,\mathcal{G}(T_1+T_2-\tau_3-\tau_2)\,\mathcal{G}(T_1+T_2-\tau_3-\tau_2-\tau_1)\,e^{-i\omega_c(T_2-\tau_3+\tau_1)},\\ S_{\mathrm{SE,R}} = \mathcal{R}\int_{-\infty}^{\infty} dT_2 e^{i\Omega T_2}\iiint_0^\infty d[\tau_3,\tau_2,\tau_1]\,\langle\Psi_g^{(0)}|\,\hat{V}U^\dagger(\tau_1+\tau_2)\hat{V}^\dagger U^\dagger(\tau_3)\hat{V}U(\tau_2+\tau_3)\hat{V}^\dagger\,|\Psi_g^{(0)}\rangle\\ \times\mathcal{G}(T_2-\tau_3)\,\mathcal{G}(T_1+T_2-\tau_3-\tau_2)\,\mathcal{G}(T_1+T_2-\tau_3-\tau_2-\tau_1)\,e^{-i\omega_c(T_2-\tau_3+\tau_1)}.\end{aligned} \tag{57}$$

Here $\mathcal{G}(t_0) = \exp\Big[-\frac{(t-t_0)^2}{\sigma^2}\Big]$ is the Gaussian envelop with linewidth σ and central time t_0. Use the frequency-domain pulse shape:

$$\mathcal{G}(t) = \int_{-\infty}^{\infty} d\omega\;\tilde{\mathcal{G}}(\omega)\;e^{i\omega t} \tag{58}$$

to recast the rephasing signals:

$$\begin{aligned}S_{\mathrm{GSB,R}}(\Omega,T_1) &= \mathcal{R}\int d[T_2,\tau_3,\tau_2,\tau_1,\omega_1,\omega_2,\omega_3]\;\langle\Psi_g^{(0)}|\,\hat{V}U^\dagger(\tau_1)\hat{V}^\dagger U^\dagger(\tau_2+\tau_3)\hat{V}U(\tau_3)\hat{V}^\dagger\,|\Psi_g^{(0)}\rangle\\ &\times\tilde{\mathcal{G}}(\omega_1)\,\tilde{\mathcal{G}}(\omega_2)\,\tilde{\mathcal{G}}(\omega_3)\,e^{i(\omega_1+\omega_2+\omega_3-\omega_c+\Omega)T_2+i(\omega_2+\omega_3)T_1+i(-\omega_1-\omega_2-\omega_3+\omega_c)\tau_3+i(-\omega_2-\omega_3)\tau_2+i(\omega_3-\omega_c)\tau_1}\\ &= \mathcal{R}\int d[\tau_2,\tau_1,\omega_1,\omega_2]\;\langle\Psi_g^{(0)}|\,\hat{V}U^\dagger(\tau_1)\hat{V}^\dagger U^\dagger(\tau_2)\hat{V}(\Omega)\hat{V}^\dagger\,|\Psi_g^{(0)}\rangle\\ &\times\tilde{\mathcal{G}}(\omega_1)\,\tilde{\mathcal{G}}(\omega_2)\,\tilde{\mathcal{G}}(\omega_c-\Omega-\omega_1-\omega_2)\,e^{i(\omega_c-\Omega-\omega_1)(T_1-\tau_2)+i(-\Omega-\omega_1-\omega_2)\tau_1}\\ &\approx \mathcal{R}\int d[\tau_2,\tau_1,\omega_1,\omega_2]\;\langle\Psi_g^{(0)}|\,\hat{V}U^\dagger(\tau_1)\hat{V}^\dagger U^\dagger(\tau_2-\tau_1)\hat{V}(\Omega)\hat{V}^\dagger\,|\Psi_g^{(0)}\rangle\\ &\times\tilde{\mathcal{G}}(\omega_1)\,\tilde{\mathcal{G}}(\omega_2)\,\tilde{\mathcal{G}}(\omega_c-\Omega-\omega_1-\omega_2)\,e^{i(\omega_c-\Omega-\omega_1)(T_1-\tau_2)+i(-\Omega)\tau_1}\\ &= \mathcal{R}\int d[\tau_2,\omega_1,\omega_2]\;\langle\Psi_g^{(0)}|\,\hat{V}\hat{V}^\dagger(-\Omega)U^\dagger(\tau_2)\hat{V}(\Omega)\hat{V}^\dagger\,|\Psi_g^{(0)}\rangle\\ &\times\tilde{\mathcal{G}}(\omega_1)\,\tilde{\mathcal{G}}(\omega_2)\,\tilde{\mathcal{G}}(\omega_c-\Omega-\omega_1-\omega_2)\,e^{i(\omega_c-\Omega-\omega_1)(T_1-\tau_2)}\\ &= \mathcal{R}\sum_{\boldsymbol{\nu}}|\,\langle\Psi_g^{(\boldsymbol{\nu})}|\,\hat{V}(\Omega)\hat{V}^\dagger\,|\Psi_g^{(\mathbf{0})}\rangle\,|^2 L^{\boldsymbol{\nu}}_{\mathrm{GSB,R}}(T_1;\omega_c,\Omega)\end{aligned} \tag{59}$$

where:

$$L^{\boldsymbol{\nu}}_{\mathrm{GSB,R}}(T_1;\omega_c,\Omega) = \int d\omega_2\tilde{\mathcal{G}}(\Omega-\omega_c-E_g^{\boldsymbol{\nu}})\,\tilde{\mathcal{G}}(\omega_2)\,\tilde{\mathcal{G}}(\omega_2+E_g^{\boldsymbol{\nu}})e^{iE_g^{\boldsymbol{\nu}}T_1} \tag{60}$$

Here the approximation holds well while terms with $\omega_1 + \omega_2 \approx E_g^{\boldsymbol{\nu}}$ dominates the signal. Similarly one can obtain the rephasing SE signals:

$$
\begin{aligned}
S_{\mathrm{SE,R}}(\Omega, T_1) &= \mathcal{R} \int d[T_2, \tau_3, \tau_2, \tau_1, \omega_1, \omega_2, \omega_3]\, \langle \Psi_g^{(0)} |\, \hat{V} U^\dagger(\tau_1 + \tau_2) \hat{V}^\dagger U^\dagger(\tau_3) \hat{V} U(\tau_2 + \tau_3) \hat{V}^\dagger \,| \Psi_g^{(0)} \rangle \\
&\times \tilde{\mathcal{G}}(\omega_1)\, \tilde{\mathcal{G}}(\omega_2)\, \tilde{\mathcal{G}}(\omega_3)\, e^{i(\omega_1+\omega_2+\omega_3-\omega_c+\Omega)T_2 + i(\omega_2+\omega_3)T_1 + i(-\omega_1-\omega_2-\omega_3+\omega_c)\tau_3 + i(-\omega_2-\omega_3)\tau_2 + i(\omega_3-\omega_c)\tau_1} \\
&= \mathcal{R} \int d[\tau_2, \tau_1, \omega_1, \omega_2]\, \langle \Psi_g^{(0)} |\, \hat{V} U^\dagger(\tau_1 + \tau_2) \hat{V}^\dagger \hat{V}(\Omega) U(\tau_2) \hat{V}^\dagger \,| \Psi_g^{(0)} \rangle \\
&\times \tilde{\mathcal{G}}(\omega_1)\, \tilde{\mathcal{G}}(\omega_2)\, \tilde{\mathcal{G}}(\omega_c - \Omega - \omega_1 - \omega_2)\, e^{i(\omega_c-\Omega-\omega_1)(T_1-\tau_2) + i(-\Omega-\omega_1-\omega_2)\tau_1} \\
&\approx \mathcal{R} \int d[\tau_2, \tau_1, \omega_1, \omega_2]\, \langle \Psi_g^{(0)} |\, \hat{V} U^\dagger(\tau_1 + \tau_2) \hat{V}^\dagger U(\tau_1) \hat{V}(\Omega) \hat{V}^\dagger \,| \Psi_g^{(0)} \rangle \\
&\times \tilde{\mathcal{G}}(\omega_1)\, \tilde{\mathcal{G}}(\omega_2)\, \tilde{\mathcal{G}}(\omega_c - \Omega - \omega_1 - \omega_2)\, e^{i(\omega_c-\Omega-\omega_1)(T_1-\tau_2) + i(-\Omega)\tau_1} \\
&= \mathcal{R} \int d\tau_2 \sum_{\boldsymbol{\nu}} |\, \langle \Psi_g^{(\boldsymbol{\nu})} |\, \hat{V}(\Omega) U(\tau_2) \hat{V}^\dagger \,| \Psi_g^{(\mathbf{0})} \rangle \,|^2 \mathcal{G}(\tau_2 - T_1)\, \tilde{\mathcal{G}}(\omega_c - \Omega - E_g^{\boldsymbol{\nu}}) \times e^{i(-\omega_c+\Omega)(\tau_2 - T_1)}
\end{aligned}
\tag{61}
$$

The final PP signals consist of at least the two parts above. Here a cleaner form of $S_{\mathrm{PP}} = \mathrm{Re}\,(S_{\mathrm{GSB}} + S_{\mathrm{ESE}})$ is provided:

$$
S_{\mathrm{SE}}(\Omega, T) \propto \sum_{\boldsymbol{\nu}} \int \mathrm{d}\tau \left| \langle \Psi_0^{(\boldsymbol{\nu})} |\, \mathrm{V}(\Omega) \mathrm{U}(\tau) \mathrm{V}^\dagger \,| \Psi_0^{(\mathbf{0})} \rangle \right|^2 \mathcal{E}(\tau - T)\, \tilde{\mathcal{E}}(\omega_c - \Omega - E_0^{\boldsymbol{\nu}}) \tag{62a}
$$

$$
S_{\mathrm{GSB}}(\Omega, T) \propto \sum_{\boldsymbol{\nu}} \left| \langle \Psi_0^{(\boldsymbol{\nu})} |\, \mathrm{V}(\Omega) \mathrm{V}^\dagger \,| \Psi_0^{(\mathbf{0})} \rangle \right|^2 e^{\mathrm{i} E_0^{\boldsymbol{\nu}} T} \mathcal{Z}^{(\boldsymbol{\nu})}(\omega_c, \Omega) \tag{62b}
$$

where $\mathcal{Z}^{(\boldsymbol{\nu})}(\omega_c, \omega) = \int \mathrm{d}\omega' \tilde{\mathcal{E}}(\omega - \omega_c - E_0^{\boldsymbol{\nu}})\, \tilde{\mathcal{E}}(\omega')\, \tilde{\mathcal{E}}(\omega' + E_0^{\boldsymbol{\nu}})$ collects the field part. One can see that eq. (62b) and eq. (62a) will yield almost indistinguishable results for comparable Ω at small T.

Experimental feasibility and testable predictions

The following files are available free of charge.

We evaluate the experimental feasibility of time-resolved quantum ghost spectroscopy (tr-QGS) for probing nonadiabatic dynamics in molecular aggregates such as PBI-1 trimers and provide specific, testable predictions to guide experimental implementation.

Photon source requirements

The entangled twin-photon state described by Eq. (2) can be generated via spontaneous parametric down-conversion (SPDC) in a nonlinear crystal such as β-barium borate (BBO). The required parameters are:

- **Pump center wavelength:** $\omega_+ = 4.7$ eV (269 nm), accessible via third- or fourth-harmonic generation from a Ti:sapphire oscillator (800 nm $\rightarrow$ 267 nm) or an Yb-doped fiber laser (1030 nm $\rightarrow$ 258 nm). Both sources are commercially available.

- **Entanglement time τ:** Determined by the inverse bandwidth of the frequency-difference correlation; $\tau = 7$–200 fs (ideally tunable) is controlled by the crystal length. For example, $\tau = 7$ fs corresponds to $\approx$0.5 mm BBO. This entanglement time can be calculated with the following function:[9]

$$
\Delta t = \left| \frac{L}{2\cos\theta_{ps}} \cdot \frac{n_e(\lambda_e, \theta_s)}{c} - \frac{L}{2\cos\theta_{pi}} \cdot \frac{n_o(\lambda_o)}{c} \right| \tag{63}
$$

Here Δt is the entanglement time, $n_e(n_o)$ is the refractive index of the extraordinary (ordinary) light. L is the length of the BBO crystal. $\lambda_e(\lambda_o)$ is the wavelength of the e-light (o-light). θ_{ps} and θ_{pi} are the

separation angels of signal and idler photons. c represents the speed of light. The refractive index of the e-light is further affected by the angle between the e-light and the optical axis of the BBO crystal:

$$n_e(\theta_s) = \frac{n_e n_o}{\sqrt{n_e^2 \sin^2(\theta_s) + n_o^2 \cos^2(\theta_s)}} \tag{64}$$

and the separation angles are achieved by $n_p = n_e \cos(\theta_{ps})$[10] with n_p the refractive index of pump pulse.

In our simulation, we take the following parameters: $n_e = 1.56,\ n_o = 1.67,\ \theta_s = 0.6,\ n_p = 1.76,\ \Delta t =$ 7 fs, $c = 3 \times 10^8$ m/s. By inserting all these parameters into eq. (63), we get the approximate length of BBO crystal as $L \approx$0.5 mm

- **Spectral correlation width σ:** $\sigma^{-1} = 400$ fs ($\approx$4 meV), standard for Type-I SPDC with a narrow-band pump.
- **Coincidence count rate:** $> 10^4$–10^5 s^{-1} is routinely achieved with 100 mW pump power and modern photon-counting detectors.

Signal detection

The tr-QGS signal requires coincidence counting of: (1) fluorescence photons emitted from the molecular sample in the signal (s) arm, and (2) idler photons (i arm) that have propagated through a variable delay line. Both arms require single-photon sensitivity with timing resolution better than τ. Commercially available options include:

- **Superconducting nanowire single-photon detectors (SNSPDs)** have exceptional specification, such as timing jitter <10 ps, detection efficiency >90% in the visible–NIR, and dark count rate <10 cps. Those are ideal detectors for the proposed experiments but require cryogenic operation ($\approx$2 K) and are relatively expensive.
- **Hybrid photomultiplier tubes (HPMTs)** offer timing jitter 50–100 ps, quantum efficiency $\approx$40% in the near-UV–visible range, room-temperature operation.
- **Silicon avalanche photodiodes (SPADs) operating in Geiger mode** typically deliver timing jitter 30–50 ps, provided quantum efficiency $\approx$50% at 500–600 nm, and allow room-temperature operation. The operating spectral range, however, might be limited due to potential sacrifices in quantum efficiency and dark noise.

The time delay T between s and i arms can be controlled by a motorized delay stage with $\leq$1 fs precision, achievable with piezo stages and interferometric stabilization. Spectral resolution is set by a grating spectrometer (e.g., Czerny–Turner with 1200 l/mm grating, resolving power $\lambda/\Delta\lambda \approx$ 2000–5000), corresponding to a spectral resolution of 0.1–0.4 eV at 500 nm.

Sample and signal strength

For PBI-1 trimers in dilute solution or thin film (optical density $\approx$0.1 at 375 nm excitation), the fluorescence quantum yield is typically >0.5. The estimated fluorescence photon flux incident on the detector is:

- Excitation: 100 μW at 375 nm (2×10^{14} photons/s)
- Absorbed photons: $\approx 10^{13}$ photons/s (assuming OD = 0.1 of the sample)
- Emitted fluorescence: 10^{12} photons/s (assuming quantum yield 0.1 or 10%)
- Collected efficiency (assuming NA = 0.22 of the collecting lens to match the optical fiber): 1%, yielding 10^{10} photons/s at the detector

- Coincidence detection efficiency (product of s and i arms): 10%, giving an estimated coincidence count rate of 10^9 s^{-1}

Even accounting for additional losses (filters, gratings, fiber coupling), count rates of 10^6–10^8 s^{-1} are readily achievable. Thus, a full tr-QGS spectrum (e.g., 100×100 pixels in ω-T space) could therefore be acquired in seconds to minutes—well within practical limits.

Tr-QGS with various σ

Here we list the figure of tr-QGS with varying σ in the entangled photon pair wave function

$$\exp\left[-(\omega_s+\omega_i-\omega_+)^2\frac{1}{\sigma^2}\right] \tag{65}$$

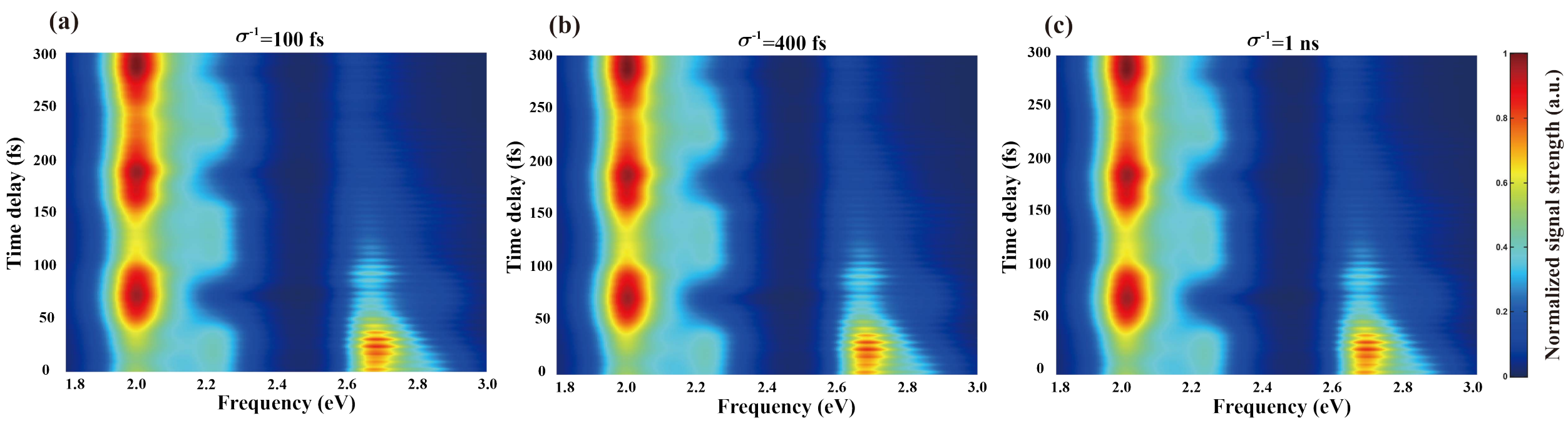

Figure 3: tr-QGS with different σ (a) $\sigma^{-1}=100$ fs, which is slightly wider in the frequency domain than signals in (b) and (c). (b) $\sigma^{-1}=400$ fs, which is the case we consider in the main text. (c) $\sigma^{-1}=1$ ns. The signal shows no difference with the situation in (b)

References

[1] Giovannetti, V. *Science* **2004**, *306*, 1330–1336.

[2] Sun, F. W.; Ou, Z. Y.; Guo, G. C. *Phys. Rev. A* **2006**, *73*, 023808.

[3] Macchiavello, C. *Phys. Rev. A* **2003**, *67*.

[4] Schlawin, F.; Gessner, M. *Quantum Sci. Technol.* **2026**, *11*, 02LT01.

[5] Liu, J.; Yuan, H.; Lu, X.-M.; Wang, X. *J. Phys. A: Math. Theor.* **2019**, *53*, 023001.

[6] Stasi, L.; Gras, G.; Berrazouane, R.; Perrenoud, M.; Zbinden, H.; Bussières, F. *Phys. Rev. Applied* **2023**, *19*, 064041.

[7] Ding, C.; Zhang, X.; Xiong, J.; Xiao, Y.; Zhang, T.; Huang, J.; Xu, H.; Liu, X.; You, L.; Wang, Z.; Li, H. *ACS Photonics* **2025**, *12*, 4924–4931.

[8] Schlawin, F. *J. Chem. Phys.* **2026**, *164*.

[9] Feng, T.; Fan, R.; Fan, R.; Wang, C.; Huang, C.; Li, A.; Zhang, H.; Cao, Y.; Li, L. *Opt. Express* **2024**, *32*, 40283.

[10] Rojpattanakul, W.; Bongkodmalee, T.; Boonkham, K. *J. Phys. Conf. Ser.* **2019**, *1380*, 012023.
